# The Impact of UVC Light on Indoor Air Chemistry: A Modeling Study

**DOI:** 10.1021/acs.est.5c07414

**Published:** 2025-07-30

**Authors:** Toby J. Carter, David R. Shaw, Ewan Eadie, Jose L. Jimenez, Paula J. Olsiewski, Zhe Peng, Charles J. Weschler, Nicola Carslaw

**Affiliations:** † Department of Environment and Geography, 8748 University of York, York YO10 5NG, U.K.; ‡ National Centre for Atmospheric Science, Department of Chemistry, University of York, York YO10 5DD, U.K.; § 1251 NHS Tayside, Photobiology Unit, Ninewells Hospital and Medical School, Dundee DD1 9SY, U.K.; ∥ Department of Chemistry, 1877 University of Colorado, Boulder, Colorado 80309, United States; ⊥ Cooperative Institute for Research in Environmental Sciences (CIRES), University of Colorado, Boulder, Colorado 80309, United States; # Center for Health Security, 1466 Johns Hopkins University, Baltimore, Maryland 21202, United States; ∇ Environmental and Occupational Health Sciences Institute, 12287 Rutgers University, Piscataway, New Jersey 08854, United States; ○ International Centre for Indoor Environment and Energy, Department of Civil Engineering, Technical University of Denmark, Kongens Lyngby 2800, Denmark

**Keywords:** photochemistry, UVC, far-UVC, indoor air chemistry, box model, INCHEM-Py

## Abstract

Germicidal ultraviolet light (GUV) is gaining attention for air disinfection, particularly following the COVID-19 pandemic. GUV air cleaning devices use 222 or 254 nm light to remove airborne and surface pathogens from indoor environments, although their impact on indoor chemistry has received limited attention. This modeling study investigates the impact of GUV light on indoor air pollutant concentrations. In a simulated, occupied classroom using a 222 nm lamp with an average room irradiance of 1 μW cm^–2^, the predicted ozone production rate was 0.33 mg h^–1^ for an air change rate of 0.5 h^–1^, leading to surface interactions with occupants and inanimate surfaces that formed secondary products including nonanal, decanal, and 4-oxopentanal. By contrast, ozone concentration increased by 0.19 mg h^–1^ at 0.5 h^–1^ in the presence of a 254 nm lamp with an average room irradiance of 15 μW cm^–2^, primarily due to infiltration. The long-term health benefits of GUV light disinfection need to be quantitatively compared to the health harms due to GUV-induced pollution to allow a more complete assessment of the benefits of this technology.

## Graphical abstract

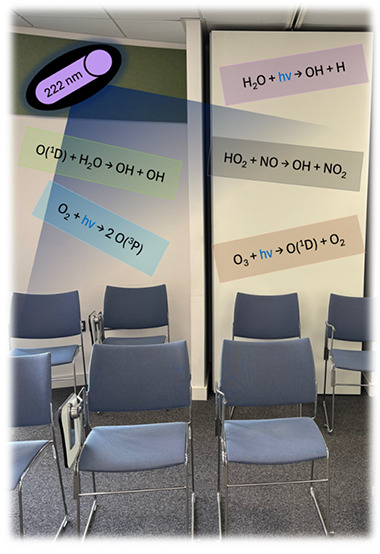

## Introduction

The COVID-19 pandemic highlighted the need for adequate ventilation to prevent transmission of viruses indoors, particularly in crowded locations like hospitals and schools. The realization that transmission of the virus was mostly airborne^1,2^ led to the development of numerous air cleaning device technologies to remove airborne and surface pathogens. Air cleaning devices have been proposed to remove indoor air pollutants, although some of the technologies can adversely affect indoor air quality and/or human health.^3–5^

Devices which emit light in the UVC region (200–280 nm) have become increasingly popular for household, office and healthcare air cleaning following the COVID-19 pandemic.^6,7^ These devices are commonly referred to as germicidal UV (“GUV”) air disinfection lamps and have peak emissions at wavelengths of 222 nm (GUV222) or 254 nm (GUV254), which can inactivate airborne viruses including COVID-19.^8–12^

A number of studies have claimed that exposure to GUV222 light (also categorized as far-UVC light (200–230 nm)) has a minimal effect on human health.^8,13–15^ Several studies have shown no acute reaction in human skin^14,16^ or eyes^15,17^ when appropriately filtered GUV222 is deployed in occupied spaces, although it can cause eye irritation if directly observed.^18^ In one of the few longer term studies in an occupied space, Sugihara et al. (2023) installed two KrCl excimer lamps in an examination room to assess the impact of extended exposure to GUV222 light.^15^ Six ophthalmologists worked in the irradiated room for approximately 6.7 h per week for a year, with an 8 h light irradiation dose of 6.4 mJ cm^–2^. No chronic or acute health effects were observed as a result of the extended GUV222 light irradiation, which also produced a >90% deactivation of microorganisms in the room.^15^

By contrast, Ong et al. (2022) found a greater degree of double-strand break DNA damage, but much less DNA damage typically associated with exposure to the sun, from GUV222 compared to GUV254 light.^19^ GUV254 light is acutely harmful to humans, causing erythema (sunburn) on exposed skin and photokeratitis in eyes^20–22^ and should be avoided in occupied sections of a room. Upper-room GUV254 has a long history of use to avoid and prevent health impacts,^23^ dating back to 1937 with efforts to prevent the spread of measles in schools.^24^ A potential advantage of GUV222 light in this respect, is that it may be harmless to human skin and eyes at irradiation levels which efficiently inactivate airborne pathogens.^25,26^

Despite the proven efficacy of pathogen removal by GUV222 and GUV254 light, the implications for indoor air chemistry have been relatively understudied. Link et al. (2023) discovered that a filtered GUV222 lamp with a fluence rate of 2.6 μW cm^–2^, produced over 50 ppb of ozone (O_3_) (steady-state) after operation for 4 h.^27^ The average ozone generation rate was 19.4 ± 0.3 ppbv h^–1^ in a 31.5 m^3^ stainless steel chamber with an air change rate (ACR) of 0.012 h^–1^. Peng et al. (2023) found that in a sealed (ACR of 0 h^–1^) 21 m^3^ Teflon reaction chamber with a filtered GUV222 lamp with a fluence rate of 2.0 μW cm^–2^, the ozone generation rate was approximately 22 ppb h^–1^, reaching 80 ppb after 4 h.^28^ These chamber studies help us to understand ozone generation rates from GUV222, but do not use typical air change rates or contain the complex mixture of internal surfaces and hence chemical interactions you would expect in real-world environments.

Ozone formed by GUV222 light creates the potential for oxidant chemistry indoors, given it leads to OH (hydroxyl radical) production.^29–33^ Both oxidants react with volatile organic compounds (VOCs) to form secondary organic aerosols (SOA) and oxygenated VOCs such as formaldehyde. By contrast, in a 110 m^3^ laboratory at air change rates of 2.8–4.2 h^–1^, Graeffe et al. (2023) found that the ozone concentration decreased by ≈2 ppb in the presence of intense GUV254 lights to ≈11 ppb, relative to a steady-state concentration of ≈13 ppb when the lamp was off.^34^ The authors also noted that, in some instances, ozone production also took place, likely from nitrogen dioxide (NO_2_) photolysis.

The aims of this paper are to (i) use an indoor air chemistry model to understand how GUV light affects indoor gas-phase chemistry in more detail than previously (ii) explore the surface production of oxygenated VOCs following ozone deposition for the first time. This builds on previous modeling work which adopted much simpler chemistry schemes^29,31,33^ and addresses the limitation of neglecting secondary chemistry, as highlighted by Barber et al. (2023).^33^ We explore the impact of different wavelengths of light on oxidant formation, and investigate the impact of GUV222 and GUV254 light in a simulated, occupied school classroom.

## Methods

### The INCHEM-Py Model

This study uses the INdoor CHEmical Model in Python (INCHEM-Py, v1.2), a chemical box model which solves a system of ordinary differential equations, predicting temporal concentrations of indoor air species.^35,36^ INCHEM-Py has been used to investigate cooking,^37^ cleaning,^38,39^ indoor-outdoor air exchange,^40^ and surface interactions.^41,42^

INCHEM-Py assumes a well-mixed environment and uses the Master Chemical Mechanism (MCM) v3.3.1,^43^ which incorporates approximately 6000 species and 20,000 reactions.^44^ It describes the atmospheric degradation of 143 VOCs including schemes for isoprene, limonene, α- and β-pinene and β-caryophyllene.^45–49^ The VOC degradation is initiated via reaction with OH,^50^ O_3_,^51^ NO_3_ ^52^ or photolysis as relevant. These reactions form hydroperoxy (HO_2_), organic peroxy (RO_2_), alkoxy (RO) radicals and Criegee intermediates (R’R“COO), which undergo further reactions until the final oxidation products are formed.^44^ INCHEM-Py considers irreversible surface deposition of 3371 gas-phase species.^36^ The model also includes a deposition mechanism for ozone and hydrogen peroxide (H_2_O_2_) onto several indoor surfaces, including soft fabrics and skin, which cause at-surface oxidation reactions and emit secondary pollutants. These surface mechanisms are explained further in detail elsewhere.^36,41,53,54^

INCHEM-Py solves eq 1, predicting indoor concentrations (*C*_*i*_) over time (*t*) in molecules cm^–3^.

1 $$\frac{dC_{i}}{dt}=\sum R_{ij}+\left(\lambda_{r}C_{i,\mathrm{out}}-\lambda_{r}C_{i}\right)-\nu_{d_{i}}\left(\frac{A}{V}\right)C_{i}+k_{t}$$

where ∑*R*_*ij*_ represents the sum of reactions between species *i* and all other species *j*, λ_*r*_ is the ACR in air changes per second (s^–1^), *C*_*i,*out_ is the outdoor concentration of species *i* (molecules cm^–3^), ν_*di*_ is the surface deposition velocity (cm s^–1^), *A* is the area of internal surfaces (cm^2^), and *V* is the volume of the indoor environment (cm^3^). The term, *k*_*t*_, refers to indoor emission rates of species *i* (molecules cm^–3^ s^–1^).

### Development of the Model

INCHEM-Py v1.2 originally considered 44 photolysis processes (Table S1), summing the transmission of light between 300 and 760 nm through windows, with one of seven artificial indoor lights to find the total indoor photolysis rate. INCHEM-Py has been developed to include photolysis between 200 to 300 nm, following methodology developed by Wang et al. (2022).^55^ The new wavelength range was split into 10 nm subregions, which are named according to the midwavelength of each 10 nm range (Table S2). For example, the UV205 label denotes irradiance data from the wavelength range between 200 ≤ λ < 210 nm.

Molecular oxygen (O_2_) absorbs light between 175 and 242 nm with a quantum yield of one,^56,57^ yielding two ground state oxygen atoms (O^3^P) (Reaction 2), which then react with oxygen molecules to produce O_3_ (Reaction 3), with M representing a molecule of air which soaks up excess energy, usually O_2_ or N_2_.^27,56–58^ Reaction 2 has been incorporated into INCHEM-Py: reaction 3 was already included.

R1 $$O_{2}+h\nu \overset{175\;nm\;<\;\lambda \;<\;242\;\mathrm{nm}}{\to }2O{(}^{3}P)$$

R2 $$O{(}^{3}P)+O_{2}+M\to O_{3}+M$$

The photolysis of water vapor was also added to INCHEM-Py using the absorption crosssection and quantum yield data from Ranjan et al. (2020)^59^ (Reaction 4, Table S1).

R3 $$H_{2}O+h\nu \overset{190\mathrm{nm}<\lambda <230\mathrm{nm}}{\to }OH+H$$

The photolysis rate coefficient for each individual species between 200 and 300 nm is calculated using eqs 5 and 6.

2 $$j_{uvc}=h_{uvc}\left(\lambda_{200}^{300}\right)I_{uvc}\left(\lambda_{200}^{300}\right)$$

where:

3 $$h_{uvc}\left(\lambda_{200}^{300}\right)={\left(100\mathrm{nm}\right)}^{-1}\int_{200\mathrm{nm}}^{300\mathrm{nm}}\sigma \phi d\lambda$$

Where *j*_*uvc*_ is the photolysis coefficient (in s^–1^), *h*_*uvc*_ is the product of the quantum yield (ϕ) (dimensionless) and the absorption cross-section (σ) in cm^2^ for that wavelength range and *I*_*uvc*_ is the spherically integrated photon flux (photons cm^–2^ s^–1^).^55^

The absorption cross-sections and quantum yields (at 298 K where possible) between 200–300 nm were obtained from literature.^43,60,61^ The spherically integrated photon flux values were calculated using spectral irradiance values measured 20 cm from a KrCl excimer lamp by Eadie et al. (2022)^9^ focused on 222 nm. The spectral irradiance of the lamp is given in Figure S1. Irradiance was converted to a photon flux using eq 7:

4 $$I=\frac{R}{E}$$

where:

5 $$E=\frac{hc}{\lambda }$$

where *R* is the irradiance (μW cm^–2^), *E* is the energy of the photon (J), *h* is Planck’s constant (J s), *c* is the speed of light (m s^–1^) and λ is the wavelength (m).

The photolysis coefficients in each interval were then calculated using eqs 5 and 6 for 10 nm wavelength ranges between 200 and 300 nm (UVC bins).

## Model Simulations and Assumptions

### Model Evaluation

Before carrying out model simulations to explore the impacts of GUV on indoor air chemistry, the model was tested against measured ozone concentrations collected by Peng et al. (2023) in a 33 m^3^ office with a GUV222 lamp.^28^ There were no occupants in the office during the measurements and a constant external ozone concentration of 3 ppb was assumed based on Peng et al. (2023).^28^ Measured ACRs ranged from 0.62 to 0.96 h^–1^,^28^ so we used the median value of 0.79 h^–1^. One GUV222 lamp (Ushio B1) was placed in the office, which had an average room irradiance of 0.86 μW cm^–2^ (photon flux of 8.7 × 10^11^ photons cm^–2^ s^–1^) and was switched on and off every 3 h. The photolysis coefficients were calculated according to the methodology described above and are given in Table S3. Temperature and relative humidity were assumed to be 295.7 K and 50%, respectively. We assumed a surface area-to-volume ratio of 1.0 m^–1^ (with an internal surface area of 33 m^2^) and that ozone and hydrogen peroxide deposited onto all surfaces at 0.0345 cm s^–1^ and 0.045 cm s^–1^ respectively.^36^

### UVC Bin Simulations

The impact of the ten UVC bins on indoor air chemistry was explored using a simulated kitchen in a typical house in suburban London, UK, at 51.45 ° N on the 21st June 2024. The temperature, relative humidity and ACR were 19.9 °C, 53.8% and 0.5 h^–1^ respectively based on recent reviews.^62,63^ Typical outdoor mixing ratios were collated from literature (Table S4).^36^ The simulated kitchen had a total surface area of 63.3 m^2^ and a volume of 25.0 m^3^ based on Manuja et al. (2019),^64^ giving a total surface area-to-volume ratio of 2.53 m^–1^. The materials and respective surface areas in the simulated kitchen are soft fabric (2 m^2^), paint (25 m^2^), wood (17 m^2^), metal (8 m^2^), concrete (1 m^2^), paper (0.2 m^2^), plastic (7 m^2^), glass (1 m^2^)^64^ and 1 adult (2 m^2^ of skin).^54,65^ The surface chemistry of ozone and hydrogen peroxide is described in Carter et al. (2023),^41^ and the primary surface emissions for painted and wooden materials are provided in Table S5.^40^

The simulations use the photolysis rate coefficients for the ten UVC bins (Tables S6 and S7), which have been calculated based on measurements at 20 cm from a single UVC light focused on 222 nm. A baseline run was conducted with no indoor or outdoor light, and the ten 10 nm wavelength ranges between 200 and 300 nm considered individually to understand how they each affect indoor gas-phase chemistry. These ten “lamps” are effectively ten wavelength sections of the emission from a single UVC 222 nm lamp without renormalization. We assumed the simulated room was illuminated with UVC light between 07:00 and 19:00 h, with no other indoor or outdoor light.

### Occupied Classroom Simulations

In order to explore the impact of GUV light on a highly occupied indoor setting, we simulated an occupied classroom, based on Park et al. (2024).^31^ Park et al. (2024) used CFD simulations to predict ozone, OH, OVOC (oxygenated VOC) and SOA concentrations for different irradiances of GUV222 and GUV254 light and a range of ventilation rates.^31^ We wanted to explore time varying concentrations in more chemical detail than previously, as well as for a wider range of chemical species. The average room irradiances of the GUV222 lamps (1, 3, and 5 μW cm^–2^), and ACRs (of 0.125, 0.5, and 2.0 h^–1^), were based on previous studies.^27,28,31,33,66^ The average irradiances of 3 and 5 μW cm^–2^ are higher than the ACGIH (American Conference of Governmental Industrial Hygienists) guideline values for eye and skin safety,^28,67,68^ although fluence rates up to 1000 μW cm^–2^ have been proposed in some extreme cases,^69^ and our range of values allows us to understand more fully, the potential impacts on chemistry. The GUV254 lamp irradiances (of 30, 40, and 50 μW cm^–2^) were recommended by the U.S. Center for Disease Control (CDC), because they are effective in killing airborne tuberculosis pathogens.^70^ For the GUV254 lamps, Park et al. (2024)^31^ describe their room being partially irradiated, either by 15% or 30%. We have chosen to use the 30% irradiation for our GUV254 simulations, for maximum perturbation of indoor air chemistry (e.g., 50 μW cm^–2^ × 0.3 = 15 μW cm^–2^). In reality, the upper section of the room will have the highest irradiance (between 30 and 50 μW cm^–2^),^71^ however, we are assuming a well-mixed environment. The average room irradiances for the GUV254 lamp are 9, 12, and 15 μW cm^–2^ at 30% room irradiation. The photolysis coefficients for each lamp type and irradiance combination are provided in Tables S8 and S9.

The temperature and relative humidity (RH) of the classroom are 295 K and 37.5% respectively according to the simulation in Park et al. (2024).^31^ The GUV lamp is switched on at 09:00 h, when the students and teacher enter the classroom, and off at 12:00 h when they leave for lunch. The lamp is turned back on at 13:00 h after lunch, and off at 15:00 h when everyone goes home.^31^ For breath emissions, the average age of the 20 students is assumed to be 10 years old, and there is 1 adult present.^54^ Breath emissions are provided in Table S10. Outdoor mixing ratios of O_3_, nitric oxide (NO) and NO_2_ vary diurnally (Figure S2) and follow the concentration profile of suburban London, United Kingdom (latitude of 51.45° N),^36^ for June 21st 2024. We assume that no indoor artificial lighting is used with the GUV lamps and that the classroom windows contain low emissivity glass.^55^ The volume of the simulated classroom is 178 m^3^ and the spatial and compositional representation of surfaces follows Kruza and Carslaw (2019).^54^ The students and adults have 1 m^2^ and 2 m^2^ of skin surface respectively,^65^ yielding a total skin surface area of 22 m^2^. The other surfaces assumed to be present are painted walls (144 m^2^), wood (26 m^2^) and linoleum (60 m^2^).^64^ Primary surface emissions for painted and wooden materials^40^ in the classroom have been included in these simulations (Table S5). The baseline and the other simulation parameters for the classroom are provided in Table 1.

**Table 1:** Simulation Conditions for the Classroom Analysis^a^

Simulation Number	ACR (h^–1^)	Lamp Type (nm)	Average Room Irradiance (μW cm^–2^)
Low ACR Baseline	0.125	-	-
1	0.125	222	1
2	0.125	222	3
3	0.125	222	5
4	0.125	254	9
5	0.125	254	12
6	0.125	254	15
Mid ACR Baseline	0.5	-	-
7	0.5	222	1
8	0.5	222	3
9	0.5	222	5
10	0.5	254	9
11	0.5	254	12
12	0.5	254	15
High ACR Baseline	2.0	-	-
13	2.0	222	1
14	2.0	222	3
15	2.0	222	5
16	2.0	254	9
17	2.0	254	12
18	2.0	254	15
19	0.125	222	5
20	0.5	222	5
21	2.0	222	5

a For the baseline and simulations 1–18, one adult and 20 children are present. The classroom in simulations 19–21 is unoccupied. The GUV254 irradiance values assume that 30% of the room volume is illuminated by 254 nm light.

## Results

### Model Validation

To evaluate the UVC photolysis framework, ozone measurements made in an office with a GUV222 lamp by Peng et al. (2023) were simulated using INCHEM-Py. The comparison between experimental and simulated ozone measurements is shown in Figure 1.

**Figure 1:**
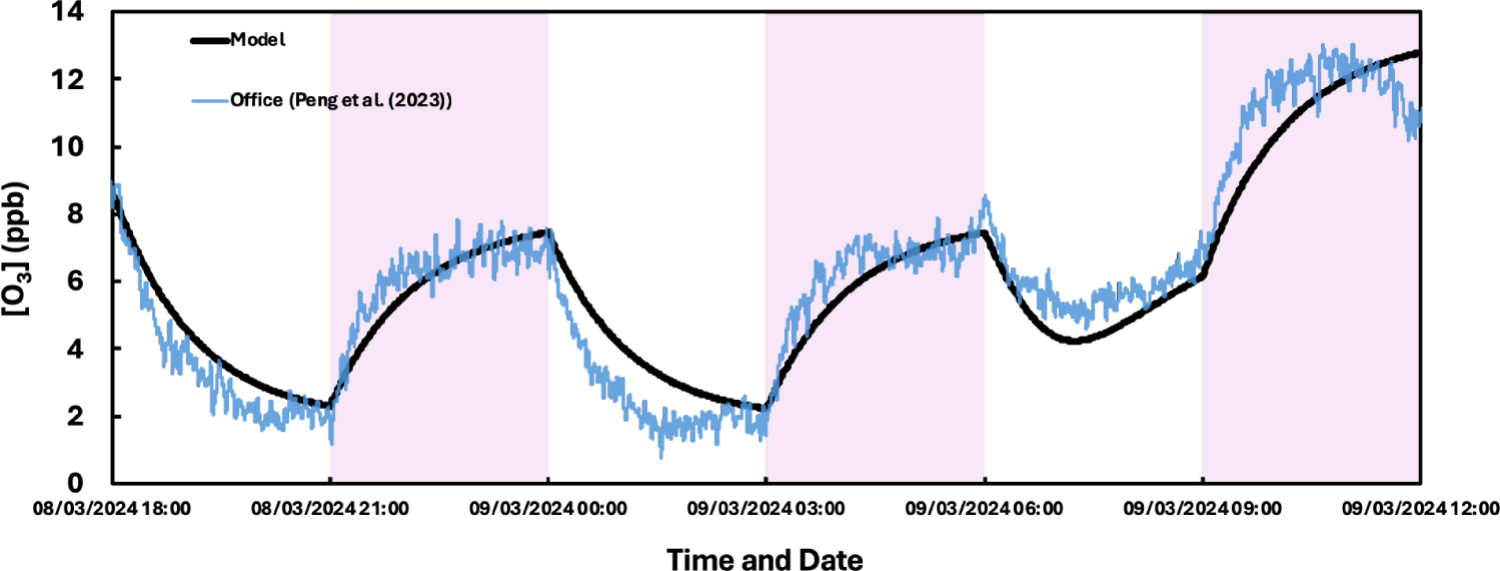
Measured (blue line) and modeled (black line) ozone concentration (ppb) in an office as a GUV222 lamp is switched on (purple area) and off (white area). The measured ozone concentrations are from Figure 1c in Peng et al. (2023).^28^

The modeled ozone concentrations compare well to those measured, reproducing the ozone increase when the lamp is switched on, and the decay when it is turned off. The simulated ozone formation rate from 21:00 to 22:00 h is 3.2 ppb h^–1^, which compares reasonably well to the experimental value (4.5 ppb h^–1^).^28^ The simulated ozone loss was 1.1 h^–1^, compared to the measured value of 0.78 h^–1^ reported in Peng et al. (2023).^28^ This level of agreement provides confidence that the model can replicate ozone production and is suitable for analysis of the subsequent indoor air chemistry initiated by GUV222 lamps.

### The Impact of UVC Wavelength on Indoor Pollutant Concentrations

Indoor concentrations of key species for different UVC wavelength ranges in a simulated kitchen are shown in Figure 2. The UVC lights were switched on at 07:00 h and off at 19:00 h with no occupant activities (cooking or cleaning), and no other light sources used (see Methods). Scenarios with incandescent lights and in darkness are included for comparison. The average indoor concentrations of key species with lights on, are provided in Table S11.

**Figure 2:**
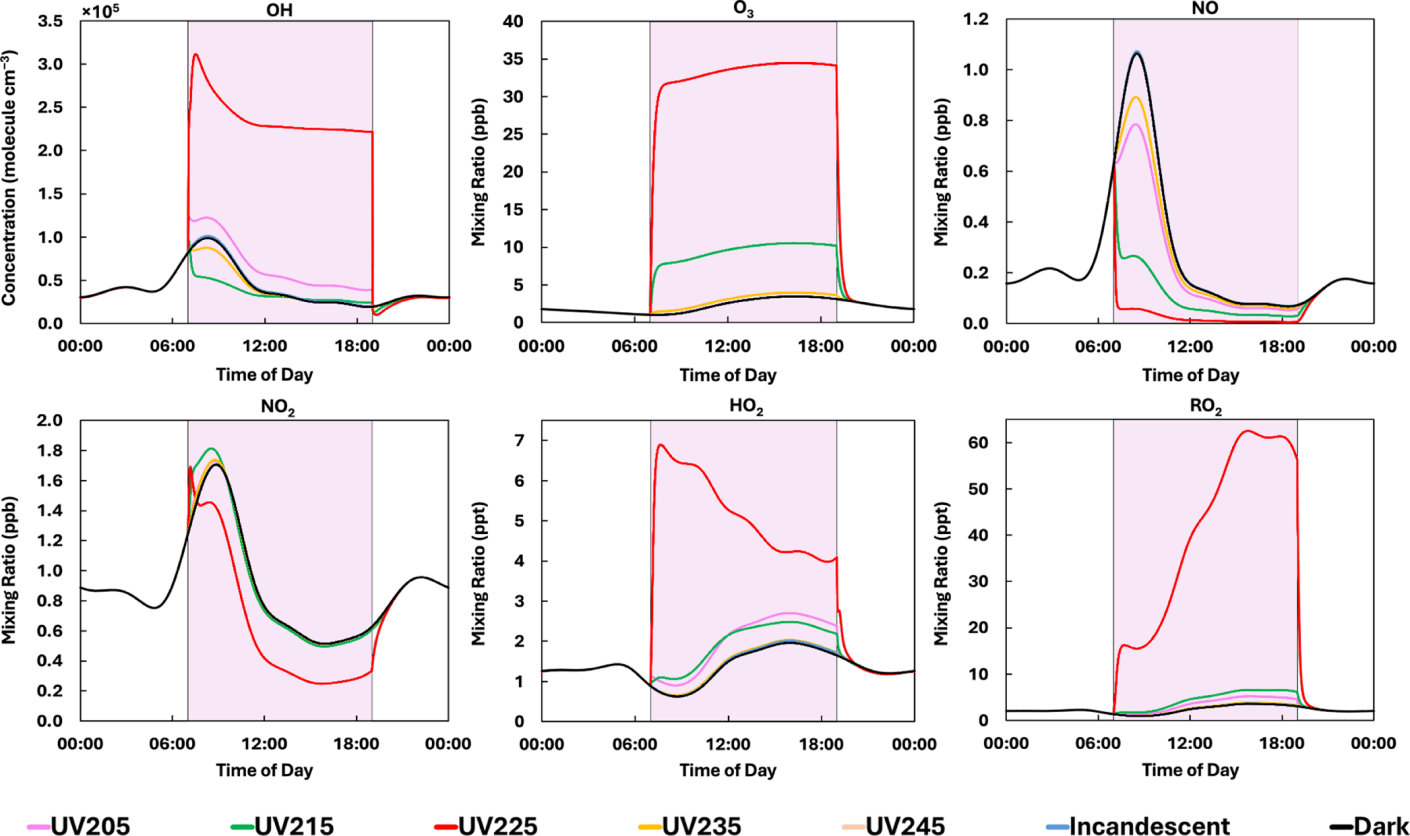
Simulated indoor concentrations in a kitchen (with a single adult present) for the five UVC light bins between 200 and 250 nm (simulations in the 250–300 nm wavelength range are excluded, as they are similar to the 240–250 nm simulation), incandescent lighting only, and in the dark. The shaded purple areas indicate when lights were turned on (07:00 h) and off (19:00 h).

The UV225 bin (effectively a GUV222 lamp) has the most significant impact on indoor concentrations. When the light was switched on, the steady-state ozone concentration increased to 31.3 ppb (from 1.0 ppb) by 07:45 h. The rate of increase (40.4 ppb h^–1^) exceeds that measured by Link et al. (2023)^27^ (19.4 ppbv h^–1^) and Peng et al. (2023)^28^ (22 ppb h^–1^) at the same wavelength, but our lamp is more intense (fluence rate of 93.8 μW cm^–2^) than used in these two studies (fluence rates of 2.6 μW cm^–2^ and 2.0 μW cm^–2^ respectively). Ozone generation below 242 nm is driven by the photolysis of oxygen (Reaction 2), with the oxygen atoms recombining with oxygen molecules (Reaction 3). The rate of Reaction 3 at 07:45 h is 139 ppb h^–1^.

After the initial rapid increase, ozone increased gradually, reaching a maximum concentration of 34.5 ppb at 16:18 h before returning to a background concentration (2.7 ppb) at 20:25 h, 1 h and 25 min after the light was switched off. The UV215 bin showed a similar trend in ozone concentration to UV225, but with a lower maximum of 10.6 ppb. The only other discernible ozone increases were for the UV205 and UV235 bins, which both increased ozone concentrations by 0.5 ppb within an hour. Above 240 nm, there was a lower average ozone concentration during the day (2.5 ppb) than with incandescent lighting, or with no artificial lighting at all (2.6 ppb).

The UV205 bin produces less chemistry than the UV225 bin, due to the higher intensity of the light in the latter. This result is not surprising as we have used irradiance data from a 222 nm lamp, so the irradiance peaks in this bin. Different wavelength lamps will produce different spectral intensities across the same wavelength range, so would need to be investigated to understand how results may differ to those reported here.

The total ozone formation rate at 07:05 h for the UV215 bin was 38.7 ppb h^–1^, much lower than for UV225 (173 ppb h^–1^) at this time. The most important ozone formation reaction at this time for the UV215 and UV225 bins is Reaction 3. Total ozone loss rates are 5.8 h^–1^ and 5.7 h^–1^ for the UV215 and UV225 bins respectively, and are dominated by surface deposition (ten times greater than ventilation loss for both scenarios).

Indoor OH concentration increased from 8.2 × 10^4^ molecules cm^–3^ to 3.1 × 10^5^ molecules cm^–3^ (a 280% increase), 30 min after the UV225 bin light was turned on. This sharp increase in OH concentration was caused by the photolysis of ozone to form excited state oxygen (O­(^1^D)) atoms (Reaction 9), followed by reaction of O­(^1^D) with water (Reaction 10) to produce OH radicals, at a rate of 0.7 ppb h^–1^ for the highest diurnal OH concentration at 07:30 h.

R4 $$O_{3}+h\nu \overset{200<\lambda <\mathrm{350 nm}}{\to }O{(}^{1}D)+O_{2}$$

R5 $$O{(}^{1}D)+H_{2}O\to OH+OH$$

For the UV215 bin, the average OH concentration (3.6 × 10^4^ molecules cm^–3^) was lower than for the other wavelength ranges tested, decreasing from 8.2 × 10^4^ to 5.9 × 10^4^ molecules cm^–3^, around 20 min after the lamp was turned on. This behavior can be explained through a consideration of ozone and OH reaction rates. Figure 3 shows the diurnal formation and loss rates for OH for the UV215 and UV225 bins. For the UV225 bin, there was a sharp increase in the OH formation rate at 07:00 h (Reaction 10). The OH produced HO_2_, which reacted with NO to recycle the OH (Figure 3). As the HO_2_ reaction with NO became less important (around 07:30 h), the OH concentration decreased, although this decrease was offset somewhat by the reaction of excited oxygen atoms with water (Reaction 10). For the UV215 bin, the loss rates outweighed the formation rates at 07:00 h, resulting in a decrease of OH (Figure 3a).

**Figure 3:**
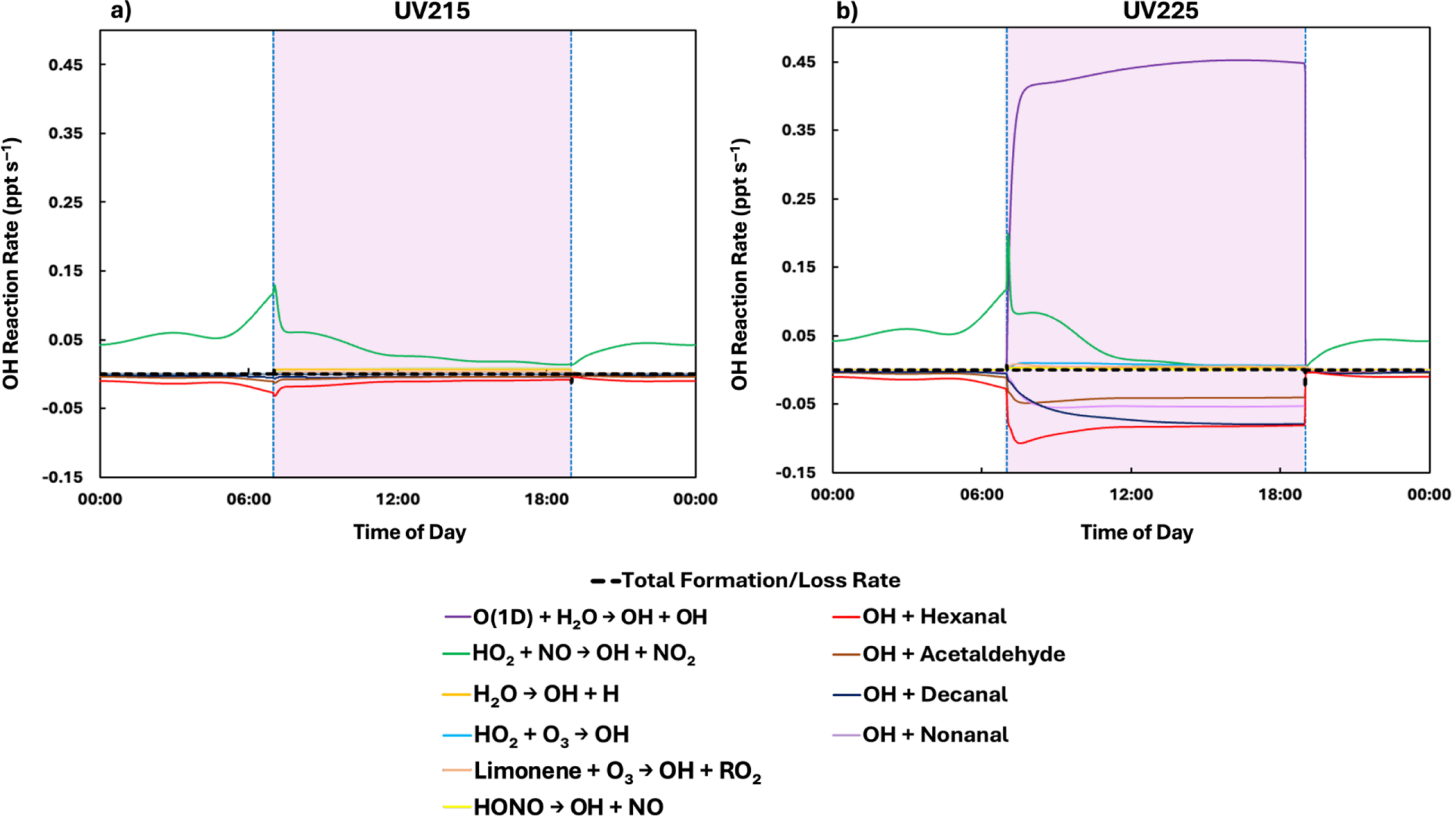
Diurnal OH formation and loss rates (ppt s^–1^) in a simulated kitchen (with a single adult present) for the UV215 bin (a) and the UV225 bin (b). The major formation and loss reactions are shown, along with the net formation/loss rate (thicker dashed black line). Dashed blue lines and the shaded purple areas indicate when lights were turned on (07:00 h) and off (19:00 h).

Due to the elevated ozone concentrations with the UV225 bin, average HO_2_ (5.1 ppt) and RO_2_ (41.3 ppt) concentrations were approximately 3.5 and 16.5 times higher respectively than with incandescent lighting (1.5 and 2.5 ppt). After the UV225 bin light was switched on, HO_2_ increased from 0.88 ppt to a peak of 6.9 ppt, before gradually decreasing until the light was switched off at 19:00 h, and returning to a background concentration of 1.5 ppt at 19:55 h. RO_2_ concentrations initially increased from 1.4 to 16.3 ppt at 07:42 h, then gradually increased to a maximum concentration of 62.5 ppt at 15:48 h, before returning to baseline levels (2.3 ppt) 95 min after the light was switched off. Enhanced oxidation reactions increased HO_2_ and RO_2_ concentrations for the UV215 bin, with average concentrations during the day of 2.0 and 4.6 ppt, respectively.

The UV215 and UV225 bins caused a decrease in NO concentration, owing to reaction with ozone. The average NO concentrations were 0.1 and 0.03 ppb, respectively. The lighting had little effect on NO_2_ concentrations, with a decrease of 0.3 ppb from the baseline value of

0.9 ppb only seen for the UV225 bin. For the UV225 bin, formaldehyde, peroxyacetylnitrates (PANs) and organic nitrates (RNO_3_, e.g., methyl nitrate, CH_3_NO_3_) had higher average diurnal indoor concentrations (9.7 ppb, 1.0 ppb, and 17.5 ppt respectively) than the other wavelength regions (see Table S11). The concentration of PANs for the other wavelength ranges was 0.5 ppb. The average organic nitrate concentration was lowest for the UV215 bin (5.6 ppt). Organic nitrates are formed from RO_2_ and NO reactions, and the product of RO_2_ and NO concentrations is lower for the UV215 bin than the other bins by almost a factor of 2.

Simulated OH reactivity increased sharply upon exposure to the UV225 bin (Figure S3) and increased steadily throughout the day. OH reactivity is defined by the sum of OH reactant concentrations multiplied by their respective rate coefficients with OH.^36,72^ While the lights were on, the OH reactivity rose from 36.0 s^–1^ to 55.8 s^–1^, with the largest increase between 07:00 h and 09:25 h (36.0 s^–1^ to 52.0 s^–1^). OH reactivity was dominated by the reaction of OH with straight-chained aldehydes the latter of which derive from ozone oxidation of indoor surfaces, primarily skin.

### Effect of Air Cleaning Devices in an Occupied Classroom

Figure 4 shows the concentrations of key indoor species, including radicals, in an occupied classroom with GUV222 or GUV254 lamps with irradiances as described in Table 1. The classroom had an ACR of 0.5 h^–1^ (simulations 7–12). Concentration profiles in the classroom for ACRs of 0.125 and 2.0 h^–1^ are given in Figures S4 and S5. Outdoor mixing ratios of ozone vary diurnally and follow a profile based on suburban London (see Figure S2).^36^

**Figure 4:**
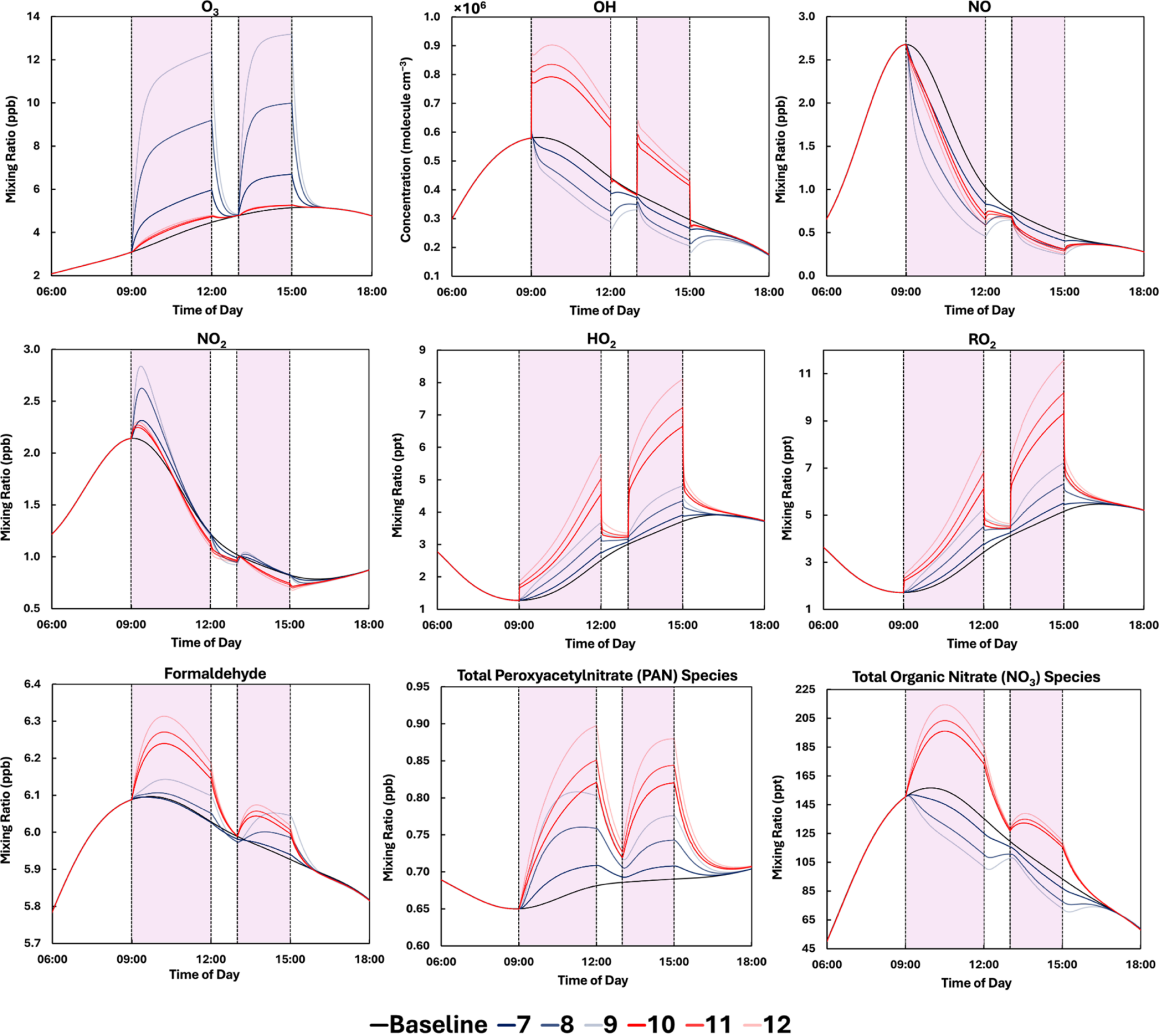
Diurnal concentrations in the simulated classroom at an ACR of 0.5 h^–1^ with GUV222 or GUV254 lamps, one teacher and 20 students. Simulations 7–9 use a GUV222 lamp with an average room irradiance of 1, 3, and 5 μW cm^–2^ respectively. Simulations 10–12 use a GUV254 lamp with an average room irradiance of 9, 12, and 15 μW cm^–2^ respectively. The baseline simulation had no lamp present, and all simulations had attenuated outdoor lighting. The lamps are on from 09:00–12:00 h, and from 13:00–15:00 h, as indicated by the purple areas.

Ozone mixing ratios increased by 1.4, 5.5, and 7.9 ppb with GUV222 lamps with average irradiances of 1, 3, and 5 μW cm^–2^ respectively, owing to “residual” ozone (generated by GUV222 but not consumed by chemistry). Some of the ozone generated by GUV222 is lost through indoor chemistry (primarily surface reactions). GUV254 lamps increase ozone mixing ratios to a lesser extent. Although ozone is photolyzed at 254 nm, most of it reforms immediately, and the ≈10% remaining forms OH which is a stronger oxidant than O_3_. If there was any net loss of O_3_ due to 254 nm light, it is replenished by what comes in from outdoors under our model conditions (outdoor O_3_ concentration ranges between 15 and 40 ppb). There is therefore a small overall increase between 09:00–12:00 h and 13:00–15:00 h corresponding to increases in outdoor ozone. The GUV254 lamp elevated the OH concentration, whereas the GUV222 lamp decreased OH concentration. The stronger the lamp power, the larger the perturbation from the baseline level, with the GUV254 15 μW cm^–2^ irradiance increasing the OH concentration by 64% at an ACR of 0.5 h^–1^. There was also an increase in OH when relative humidity was increased from 37.5% to 60% for both GUV222 (<2 × 10^4^ molecules cm^–3^) and GUV254 (≈1 × 10^5^ molecules cm^–3^) in simulations 9 and 12 respectively (Figure S6). In the case of indoor ozone, surface chemistry dominates its indoor chemistry.^73^ In the case of OH, gas-phase chemistry dominates its indoor chemistry, as is apparent from the total OH reactivity in indoor air compared to its surface removal rate.

For total organic nitrates, the GUV222 lamp decreased the mixing ratio and the GUV254 lamp increased it. The concentration of NO decreased following exposure to the GUV lamps, as enhanced ozone depleted NO. NO_2_ concentrations increased following initial GUV exposure, but by less than 1 ppb (5 μW cm^–2^ GUV222 lamp). HO_2_, RO_2_, formaldehyde and PAN mixing ratios increased following GUV light exposure, with the 15 μW cm^–2^ GUV254 lamp producing the highest concentrations.

However, the fact that ozone concentrations are only modestly elevated during lamp use does not mean there is no cause for concern. Increases in ozone, even at low concentrations, may drive significant increases in negative health effects such as asthma exacerbation^74^ and mortality.^75^ Previous work has shown that the vast majority of indoor ozone is deposited on internal surfaces.^41,53,73,76,77^ The chemical detail inherent in INCHEM-Py has allowed us to explore surface interactions following lamp use in greater detail than in previous studies. Figure 5a,b show the change in mixing ratio of a subset of surface-derived oxidative products in the occupied classroom for our simulations (note that the model simulations do not account for all oxidation products (in air and on surfaces) produced by ozone chemistry in the classroom). The ozone-surface reactions are the reason why the ozone increase induced by GUV lamps often appear to be modest,^78^ but these secondary products can also have deleterious health effects. Figure 5c shows the concentration changes without occupants.

**Figure 5:**
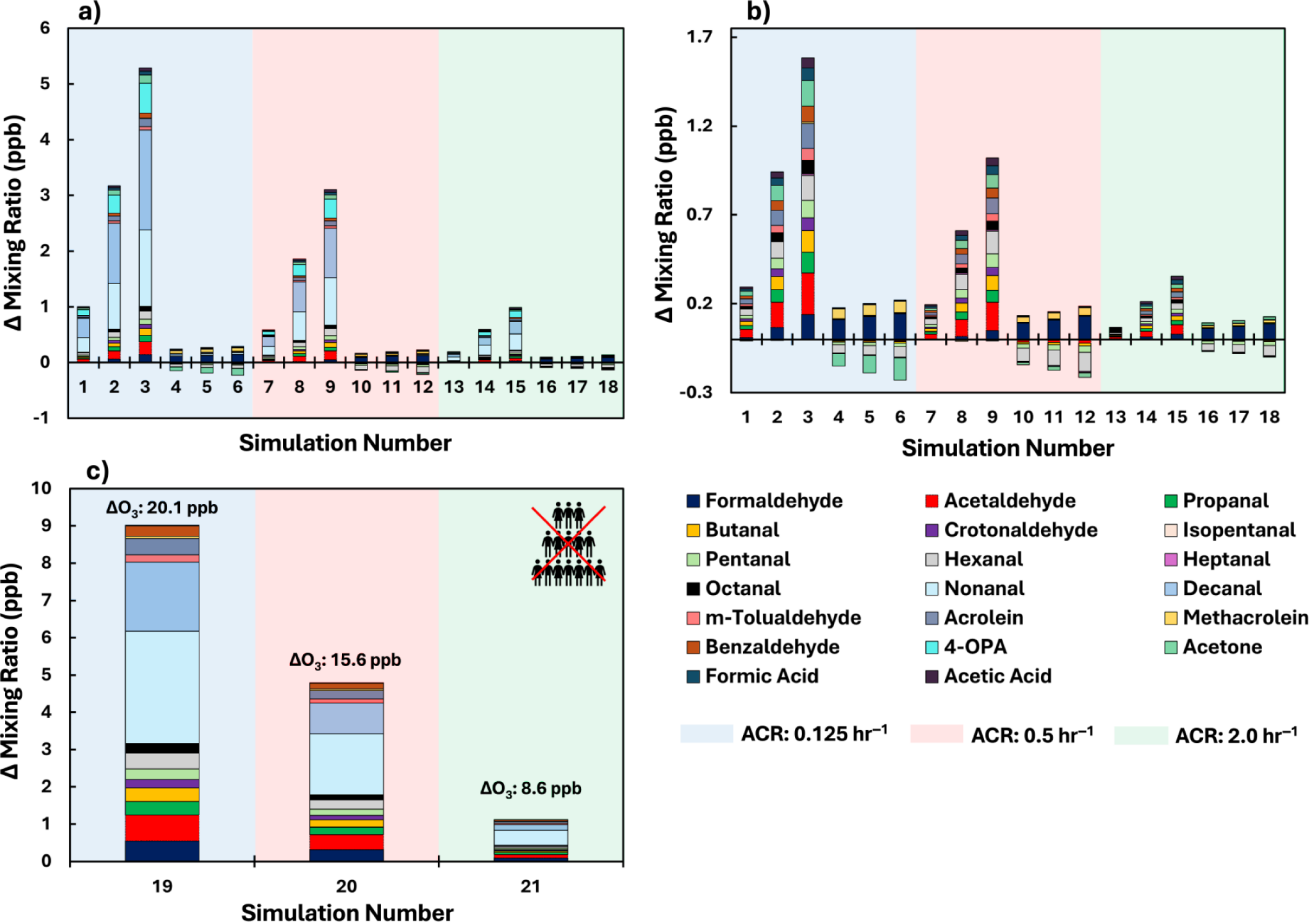
Change in mixing ratio of surface-derived oxidative products in a simulated classroom with and without GUV lamps for the 21 model simulations (see Table 1). (a) All surface-derived species. (b) Expanded view of surface-derived species without nonanal, decanal, and 4-oxopentanal. (c) Without occupants, showing the difference with and without the 5 μW cm^–2^ GUV222 lamp on. The difference in ozone concentration with and without the lamp is also shown above each bar. Simulations 19, 20, and 21 are equivalent to 3, 9, and 15, but without occupants.

Figure 5a shows that the highest increase of surface-derived oxidation products arises from a GUV222 lamp with an irradiance of 5 μW cm^–2^ at the lowest ACR of 0.125 h^–1^ (simulation 3). Nonanal, decanal and 4-oxopentanal (4-OPA) were the highest contributors to total surface oxidation product mixing ratios, contributing 1.4, 1.8, and 0.5 ppb respectively to the total. Formaldehyde and acetaldehyde were mainly emitted following ozone reactions on wooden surfaces, with nonanal, decanal and 4-OPA deriving from oxidation of constituents of skin surface lipids.^65,79^ Nonanal is also emitted when ozone reacts with carpet fibers and kitchen surfaces soiled with cooking oil.^80,81^ In the classroom with the GUV254 lamp and at all ACRs, the mixing ratios of acetone, pentanal and hexanal all decreased relative to the classroom without the lamp. A decrease in acetone concentration was observed in a previous investigation of GUV254.^82^

Ozone mixing ratios were approximately three times higher without occupants compared to with occupants (Figure S7), owing to effective uptake onto the skin surface when people are present.^83^ Because less ozone deposited onto skin surfaces in the unoccupied simulations, it was available to react on other surfaces (wood, linoleum and painted surfaces), which also produce secondary products. Some secondary products only derive from people in our simulations, so the concentrations of 4-OPA, formic acid and acetic acid are all higher in the presence of occupants (Figure 5a), especially in the low ACR setting. However, our simulations show that decanal mixing ratios are highest in the unoccupied classroom, despite this compound being a major product when ozone reacts with skin oil.^79,84^ This observation is driven by the decanal yields we use in the model, which are based on measurements of decanal emissions from painted walls and linoleum.^81^ There are no obvious chemical degradation mechanisms for paints or linoleum to produce decanal, so these measurements could reflect skin oil contamination on the tested samples. Consequently, the decanal concentrations observed in the unoccupied classroom likely represent emissions from soiled building materials, rather than direct emissions from the building materials themselves. Clearly, more measurements of emission yields from building materials would be beneficial in this respect. Table 2 shows the total loss rate (TLR) of ozone from all loss routes in the model at peak ozone concentration, and the total ozone production rate from all sources (PR) for each simulation averaged between 09:00–12:00 h. Note that PR also includes net infiltration from outdoor ozone. The distribution of ozone loss rates to surfaces, by photolysis, reactions with NO_*x*_ (NO + NO_2_), VOCs and ventilation are also given in Table 2. The net loss rates of ozone (h^–1^) through chemistry (O_3_ LR_Chemistry_) and ventilation (O_3_ LR_Ventilation_) are given in Table S12, where O_3_ LR_Chemistry_ includes loss to surfaces, photolysis, VOCs and NO_*x*_.

**Table 2:** Change in SPCP (ΔSPCP_mod_) (ppb), O_3_ PR (mg h^–1^), O_3_ TLR (h^–1^), and the Distribution of Ozone Loss in a Classroom (%) to Surfaces (*L*_Surf_), Photolysis (*L*_Photolysis_), Reaction with NO_*x*_ $\left(L_{{NO}_{x}}\right)$ , Reaction with VOCs (*L*_VOCs_) and Ventilation (*L*_Vent_)

Simulation Number	O_3_ PR (mg h^–1^)	O_3_ TLR (h^–1^)	*L*_Surf _%	*L*_Photolysis_ %	$L_{{NO}_{x}}$ %	*L*_VOCs_ %	*L*_Vent_ %	ΔSPCP_mod_ (ppb)
1	0.24	4.31	86.4	3.3	6.8	0.6	2.9	2.1
2	0.66	4.20	88.6	3.9	4.0	0.6	3.0	6.8
3	1.07	4.16	89.4	4.3	2.7	0.5	3.0	11.2
4	0.05	4.73	78.6	13.9	4.3	0.5	2.6	0.3
5	0.05	4.80	77.5	15.7	3.7	0.5	2.6	0.3
6	0.05	4.94	75.3	18.5	3.1	0.5	2.5	0.4
7	0.33	4.97	74.8	2.9	12.0	0.2	10.1	1.7
8	0.71	4.84	76.8	3.4	9.3	0.2	10.3	5.2
9	1.08	4.77	78.0	3.8	7.5	0.2	10.5	8.7
10	0.19	5.34	69.6	12.3	8.5	0.2	9.4	0.3
11	0.19	5.40	68.8	13.9	7.8	0.2	9.3	0.4
12	0.20	5.53	67.3	16.6	6.9	0.2	9.0	0.4
13	0.68	6.65	55.9	2.2	11.7	0.1	30.1	1.1
14	0.96	6.59	56.5	2.5	10.6	0.1	30.4	3.3
15	1.23	6.54	56.9	2.8	9.6	0.1	30.6	5.5
16	0.58	7.03	52.9	9.4	9.1	0.2	28.5	0.3
17	0.58	7.10	52.4	10.6	8.7	0.2	28.2	0.4
18	0.59	7.22	51.5	12.7	8.0	0.2	27.7	0.4
19	3.41	1.41	74.4	12.9	3.6	0.2	8.9	28.5
20	2.95	1.91	54.8	9.5	9.3	0.3	26.2	20.1
21	2.35	3.64	28.7	5.0	11.2	0.2	54.9	9.6

The change in secondary product creation potential (SPCP) for each simulation (averaged between 09:00–15:00 h) is also given in Table 2. SPCP is a metric to assess the production of pollutants which may impact on human health.^85^ A modified version of the SPCP (ΔSPCP_mod_) considers the sum of secondary pollutants derived from GUV-initiated chemistry (eq 11), having subtracted the baseline run with lamps turned off.

$$\Delta {\mathrm{SPCP}}_{\mathrm{mod}}=\sum \left([\mathrm{total}\;\mathrm{organic}\;\mathrm{nitrates}]+[\mathrm{total}\;\mathrm{peroxyacetylnitrates}]+\left[\mathrm{ozone}\right]+[\mathrm{glyoxal}]+\left[\mathrm{formaldehyde}\right]+\left[\mathrm{acetaldehyde}\right]+\left[\mathrm{acrolein}\right]+\left[\mathrm{propanal}\right]+\left[\mathrm{butanal}\right]+\left[\mathrm{pentanal}\right]+\left[\mathrm{hexanal}\right]+\left[\mathrm{heptanal}\right]+\left[\mathrm{octanal}\right]+\left[\mathrm{nonanal}\right]+\left[\mathrm{decanal}\right]\right)$$ 6

Many of the products of indoor ozone chemistry have unknown toxicities and some of them have only been identified within the past decade.^86–88^ The ΔSPCP_mod_ understates the impact on human health (e.g., it does not include SOA), but provides a good metric to compare potentially harmful pollutant concentrations between simulations.

Ozone loss to different sinks varies by simulation. For example, in simulation 9, 78.0% of ozone is lost onto surfaces, whereas 7.5% of ozone is lost via reaction with NO_*x*_ (predominantly reaction with NO). Photolysis (3.8%), ventilation (10.5%) and reaction with gas-phase VOCs (0.2%) account for the remainder of the ozone loss. Ozone loss through reaction with NO_*x*_ is most important (12.0% loss) in simulation 7, which has a GUV222 lamp with a 1 μW cm^–2^ irradiance assuming an ACR of 0.5 h^–1^. On average, 99% of O_3_ loss through reaction with NO_*x*_ was via NO rather than NO_2_.

In simulation 19, 0.43 ppb of acrolein is formed, which is over the CDC intermediate and chronic exposure limit.^89–91^ Simulations 2, 3, and 9 also yield increases in acrolein (0.09, 0.14, and 0.09 ppb respectively). Acrolein can be formed from ozone-surface chemistry on wood.^92^ The SPCP provides a relative measure of potentially harmful products formed through different simulations, but is clearly not a definitive measure of all harmful products that are formed. GUV222 lamps had an average SPCP (5.1 ppb) more than 14 times greater than GUV254 lamps (0.36 ppb) in occupied settings. Unoccupied classrooms had higher SPCPs, mainly driven by the higher ozone concentrations. In an occupied classroom, the highest increase in SPCP was for a GUV222 lamp with an irradiance of 5 μW cm^–2^, assuming an ACR of 0.125 h^–1^ (simulation 3). In this simulation, ozone deposited mainly onto surfaces (little is lost to outdoors), leading to the formation of secondary pollutants (Table 2). The ozone loss to occupant surfaces is approximately 2.5 times greater than to inanimate surfaces.

Sørensen et al. (2024)^93^ deployed GUV222 lamps in furnished offices and found average ozone production rates of 1040 ± 87 μg h^–1 93^ for an average ACR of 0.2 h^–1^. This value compares well to our O_3_ PR of 1.07, 1.08, and 1.23 mg h^–1^ in simulations 3, 9, and 15, respectively, in an occupied classroom during GUV222 deployment. Our O_3_ LR_Chemistry_ (1.28, 1.41, and 1.64 h^–1^) in simulations 19–21 in unoccupied classrooms also compare well to the *k*_loss_ values in unoccupied offices in Sørensen et al. (2024)^93^ (0.83–1.37 h^–1^). Our simulated ozone concentrations were lower than those observed by Link et al. (2023)^27^ and Peng et al. (2023)^28^ of 53 and 80 ppb, respectively, following 4 h exposure to a GUV222 lamp. However, these studies took place in a stainless steel and a Teflon chamber respectively, where less surface loss would be expected. In a restroom with elevated terpenoid concentrations, however, ozone concentrations only increased by 5 ppb upon GUV222 deployment.^30^ These studies appear to show an increase in ozone concentration from GUV222 lighting in “real-life” microenvironments and is in good agreement with our model results.

## Discussion

This modeling paper investigates indoor air chemistry in the presence of GUV light. Far-UVC light between 200 and 230 nm can have a significant impact on indoor gas-phase concentrations. Ozone concentrations, in our simulated kitchen, were 11 times higher than with incandescent lighting, and OH, HO_2_ and RO_2_ concentrations were also enhanced, particularly at higher irradiances. Wavelengths between 250 and 300 nm had little impact on indoor concentrations compared to incandescent lighting. Our occupied classroom simulations also demonstrated the potential for formation of oxidation products in the presence of GUV lighting indoors. Across the 18 occupied classroom simulations and for the species shown in Figure 5, the maximum concentration of oxidation products formed for GUV222 was 5.3 ppb compared to 0.08 ppb for GUV254. The oxidation products at GUV222 were predominantly nonanal and decanal, which derived from skin oil oxidation of the occupants and other internal surfaces. Enhanced ozone concentrations from UVC light can lead to increased production of secondary pollutants from surfaces, some of which are potentially harmful to health.^94^ The maximum SPCP value at GUV222 was 11.2 ppb compared to 0.4 ppb at GUV254 in occupied rooms. We note that our model study does not consider SOA formation or VOC emission from surfaces, both of which can follow exposure to UVC light, and therefore underestimates the potential health impacts.^29–32,34,95^

Experimental studies investigating how far-UVC light propagates through indoor environments would strengthen modeling studies, including better understanding of the impacts of using different intensity/numbers of lamps, the transmission of the light around indoor spaces, and how surfaces degrade and emit pollutants under UV lighting. We also note the wide variation between different lamps (e.g., operating irradiance, filter use), which can make it difficult to generalize in modeling studies such as these.^96^ Standardization procedures for lamp operation would be a useful future step. We also note that there have been few studies that explore the longer-term health effects of exposure to GUV222 light, or on the potential degradation and emission from the multitude of materials we use indoors, such as plastics or wood.^15,97,98^

UVC light offers promise as a tool to mitigate airborne disease transmission. As such, it is of value in the present (e.g., health care settings) and in preparation for the future (i.e., the next pandemic). Upper room UV at 254 nm has a history of effectual use and the present modeling study suggests that it has a relatively small impact on indoor chemistry (although the fluence rate used was lower than current CDC limits), but has implications for human health with direct exposure.^99^ When GUV222 is deployed, it does not appear to cause acute responses in the skin or eyes,^15–17^ but it can initiate more chemistry than GUV254. However, the threat posed by unintended chemistry should be weighed against the potential benefit of GUV222 as an added tool to reduce the spread of disease.

The disinfection efficiency of GUV222 is currently uncertain, and would determine which of the fluence rate scenarios simulated in this paper is actually necessary to reduce disease transmission. Modeling, informed by experiments, shows us that steps can be taken to minimize both the ozone generated by UV at 222 nm and the secondary products derived from that ozone. Adequate ventilation is one of these steps. As per the conclusions from the study of Barber et al. (2023), we agree that GUV disinfection should only be deployed in properly ventilated spaces and never used as a reason to reduce ventilation rates.^33^ The simulations in the present paper indicate that in an occupied classroom, increasing the ventilation rate from 0.5 to 2 h^–1^ reduces the ozone concentration by approximately a factor of 2 and that of ozone derived products by a factor of 3. Other studies suggest that charcoal filters,^100^ catalysts,^101^ or spent coffee grounds,^102^ can be used to remove ozone and thus reduce the resulting chemistry. The more we understand the chemistry initiated by UVC lamps, the better we can judge when and where the benefits of disinfection are substantially larger than the harms of increased pollution.

## Supplementary Material



## Notes

Further details on photolysis reactions and coefficients used in this modeling study, wavelength intervals, key indoor pollutant concentrations, reactivities, emission rates, and spectral irradiance data (PDF)
