# Supplementary material for: The Impact of UVC Light on Indoor Air Chemistry: A Modeling Study
Source: Environ Sci Technol. 2025 Jul 30;59(31):16543–55. doi: 10.1021/acs.est.5c07414 (PMC12355949; doi:10.1021/acs.est.5c07414)
Supplement: Supplementary file 1 [file es5c07414_si_001.pdf]

# Supplementary Information

# The Impact of UVC Light on Indoor Air Chemistry: A Modelling Study

Toby J. Carter[1], David R. Shaw[1,2], Ewan Eadie[3], Jose L. Jimenez[4,5], Paula J. Olsiewski[6], Zhe Peng[4,5], Charles J. Weschler[7,8] and Nicola Carslaw[1,*]

[1] Department of Environment and Geography, University of York, Wentworth Way, York, YO10 5NG, United Kingdom

[2] National Centre for Atmospheric Science, University of York, York, YO10 5DD, United Kingdom

[3] NHS Tayside, Photobiology Unit, Ninewells Hospital and Medical School, Dundee, DD1 9SY, United Kingdom

[4] Department of Chemistry, University of Colorado, Boulder, Colorado 80309, United States of America

[5] Cooperative Institute for Research in Environmental Sciences (CIRES), University of Colorado, Boulder, Colorado 80309, United States of America

[6] Center for Health Security, Johns Hopkins University, Baltimore, Maryland 21202, United States of America

[7] Environmental and Occupational Health Sciences Institute, Rutgers University, Piscataway, New Jersey 08854, United States of America

[8] International Centre for Indoor Environment and Energy, Department of Civil Engineering, Technical University of Denmark, Lyngby, 2800 Kongens, Denmark

*Corresponding Email: nicola.carslaw@york.ac.uk

Summary of SI: 37 pages, 12 tables and 7 figures.

Table S1: The photolysis reactions originally included in INCHEM-Py v1.2 (Wang *et al.*, 2022; Shaw *et al.*, 2023), plus the two new reactions for oxygen ($O_2$) and water ($H_2O$).

| Label | Reaction | Wavelength Range (nm) |
|---|---|---|
| J1 | $O_3 \rightarrow O(^1D) + O_2$ | 300 - 349 |
| J2 | $O_3 \rightarrow O(^3P) + O_2$ | 300 - 349, 400 - 700 |
| J3 | $H_2O_2 \rightarrow OH + OH$ | 300 - 350 |
| J4 | $NO_2 \rightarrow NO + O(^3P)$ | 300 - 425 |
| J5 | $NO_3 \rightarrow NO + O_2$ | 586 - 640 |
| J6 | $NO_3 \rightarrow NO + O(^3P)$ | 585 - 640 |
| J7 | $HONO \rightarrow NO + OH$ | 300 - 399 |
| J8 | $HNO_3 \rightarrow NO_2 + OH$ | 300 - 350 |
| J9 | $O_2 \rightarrow 2O(^3P)$ | 200 - 242 |
| J10 | $H_2O \rightarrow OH + H$ | 200 - 230 |
| J11 | $HCHO \rightarrow H + HCO$ | 300 - 355 |
| J12 | $HCHO \rightarrow H_2 + CO$ | 300 - 360 |
| J13 | $CH_3CHO \rightarrow CH_3 + HCO$ | 300 - 330 |
| J14 | $C_2H_5CHO \rightarrow C_2H_5 + HCO$ | 300 - 330 |
| J15 | $C_3H_7CHO \rightarrow C_3H_7 + HCO$ | 300 - 364 |
| J16 | $C_3H_7CHO \rightarrow C_2H_4 + CH_3CHO$ | 300 - 364 |
| J17 | 2-Methylpropanal (IPRCHO) $\rightarrow C_2H_4 + CH_3CHO$ | 300 - 330 |
| J18 | Methacrolein (MACR) $\rightarrow CH_2 = CCH_3 + HCO$ | 300 - 395 |
| J19 | Methacrolein (MACR) $\rightarrow CH_2 = C(CH_3)CO + H$ | 300 - 395 |
| J20 | $C_5H_8O_3 \rightarrow CH_3C(CHO) = CHCH_2O + OH$ | 300 - 395 |
| J21 | $CH_3C(O)CH_3 \rightarrow CH_3CO + CH_3$ | 300 - 327 |
| J22 | Methyl Ethyl Ketone (MEK) $\rightarrow CH_3CO + C_2H_5$ | 300 - 352 |

*continued on next page*

| Label | Reaction | Wavelength Range (nm) |
|---|---|---|
| J23 | Methyl Vinyl Ketone (MVK) $\rightarrow CH_3CH = CH_2 + CO$ | 300 - 395 |
| J24 | Methyl Vinyl Ketone (MVK) $\rightarrow CH_3CO + CH_2 = CH$ | 300 - 395 |
| J31 | Glyoxal (GLYOX) $\rightarrow CO + CO + H_2$ | 300 - 355 |
| J32 | Glyoxal (GLYOX) $\rightarrow HCHO + CO$ | 300 - 415 |
| J33 | Glyoxal (GLYOX) $\rightarrow HCO + HCO$ | 300 - 445 |
| J34 | Methylglyoxal (MGLYOX) $\rightarrow CH_3CO + HCO$ | 300 - 440 |
| J35 | 2,3-Butanedione (BIACET) $\rightarrow CH_3CO + CH_3CO$ | 300 - 460 |
| J41 | $CH_3OOH \rightarrow CH_3O + OH$ | 300 - 365 |
| J51 | $CH_3ONO_2 \rightarrow CH_3O + NO_2$ | 300 - 340 |
| J52 | $C_2H_5ONO_2 \rightarrow C_2H_5O + NO_2$ | 300 - 340 |
| J53 | $n\text{-}C_3H_7ONO_2 \rightarrow n\text{-}C_3H_7O + NO_2$ | 300 - 340 |
| J54 | $i\text{-}C_3H_7ONO_2 \rightarrow CH_3C(O)CH_3 + NO_2$ | 300 - 360 |
| J55 | $t\text{-}C_4H_9ONO_3 \rightarrow t\text{-}C_4H_9O + NO_2$ | 300 - 330 |
| J56 | 2-Oxopropyl Nitrate (NOA) $\rightarrow CH_3C(O)CH_2(O) + NO_2$ | 300 - 340 |
| J57 | 2-Oxopropyl Nitrate (NOA) $\rightarrow CH_3CO + HCHO + NO_2$ | 300 - 340 |
| J70 | $Cl_2 \rightarrow Cl + Cl$ | 300 - 550 |
| J71 | $ClNO_2 \rightarrow NO_2 + Cl$ | 300 - 470 |
| J72 | $ClONO_2 \rightarrow NO_3 + Cl$ | 300 - 430 |
| J73 | $ClONO_2 \rightarrow NO_2 + Cl$ | 300 - 430 |
| J74 | $HOCl \rightarrow OH + Cl$ | 300 - 420 |
| J75 | $OClO \rightarrow O + ClO$ | 300 - 475 |
| J76 | $ClO \rightarrow O + Cl$ | 300 - 475 |
| J77 | $ClOOCl \rightarrow ClOO + Cl$ | 300 - 400 |

*continued on next page*

| Label | Reaction | Wavelength Range (nm) |
|---|---|---|
| J78 | $ClOOCl \rightarrow ClO + ClO$ | 300 - 400 |

Table S2: The ten new wavelength intervals (UVC bins) for each 10 nm interval in the wavelength range between 200 and 300 nm, based on the lamp used in Eadie *et al.* (2022).

| Label | Wavelength Interval (nm) |
|---|---|
| UV205 | $200 \leq \lambda < 210$ nm |
| UV215 | $210 \leq \lambda < 220$ nm |
| UV225 | $220 \leq \lambda < 230$ nm |
| UV235 | $230 \leq \lambda < 240$ nm |
| UV245 | $240 \leq \lambda < 250$ nm |
| UV255 | $250 \leq \lambda < 260$ nm |
| UV265 | $260 \leq \lambda < 270$ nm |
| UV275 | $270 \leq \lambda < 280$ nm |
| UV285 | $280 \leq \lambda < 290$ nm |
| UV295 | $290 \leq \lambda < 300$ nm |

Table S3: The photolysis rate coefficients ($s^{-1}$) used in the simulation of the university office in Peng *et al.* (2023). The blank values indicate no absorption occurs.

| **Label** | **Rate Coefficient** |
|---|---|
| J1 | 1.8 x $10^{-6}$ |
| J2 | 2.0 x $10^{-7}$ |
| J3 | 2.2 x $10^{-7}$ |
| J4 | 4.1 x $10^{-7}$ |
| J5 | - |
| J6 | - |
| J7 | 1.3 x $10^{-6}$ |
| J8 | 1.2 x $10^{-7}$ |
| J9 | 3.5 x $10^{-12}$ |
| J10 | 8.1 x $10^{-15}$ |
| J11 | 4.2 x $10^{-11}$ |
| J12 | 1.1 x $10^{-10}$ |
| J13 | 1.7 x $10^{-10}$ |
| J14 | 6.7 x $10^{-10}$ |
| J15 | 1.6 x $10^{-10}$ |
| J16 | 7.4 x $10^{-11}$ |
| J17 | 2.4 x $10^{-10}$ |
| J18 | - |
| J19 | - |
| J20 | - |
| J21 | 2.6 x $10^{-9}$ |
| J22 | 8.5 x $10^{-10}$ |
| J23 | - |
| | *continued on next page* |

| **Label** | **Rate Coefficient** |
|---|---|
| J24 | - |
| J31 | - |
| J32 | - |
| J33 | - |
| J34 | 1.1 x $10^{-8}$ |
| J35 | 1.1 x $10^{-8}$ |
| J41 | 1.3 x $10^{-7}$ |
| J51 | - |
| J52 | 7.9 x $10^{-7}$ |
| J53 | 9.1 x $10^{-7}$ |
| J54 | 1.1 x $10^{-6}$ |
| J55 | 9.6 x $10^{-7}$ |
| J56 | - |
| J57 | - |
| J70 | 4.4 x $10^{-10}$ |
| J71 | 2.8 x $10^{-6}$ |
| J72 | 1.7 x $10^{-6}$ |
| J73 | 1.2 x $10^{-6}$ |
| J74 | 1.0 x $10^{-7}$ |
| J75 | - |
| J76 | - |
| J77 | - |
| J78 | - |

Table S4: The constant mixing ratios of outdoor gas-phase VOCs (ppb) in INCHEM-Py (Carter *et al.*, 2023, 2024; Shaw *et al.*, 2023) and used in this study (Uchiyama *et al.*, 2015; Baudic *et al.*, 2016; Lü *et al.*, 2006; Mentese and Bas, 2020; Bari and Kindzierski, 2018; Sturaro *et al.*, 2010; Bari *et al.*, 2016; Gallego *et al.*, 2016; Brickus *et al.*, 1998; Hellén *et al.*, 2018; Hakola *et al.*, 2009; He *et al.*, 2010; Dlugokencky, 2022; Vichi *et al.*, 2016; Liu *et al.*, 2018; Li *et al.*, 2018; EEA, 2018).

| **Species** | **Outdoor Mixing Ratio (ppb)** |
|---|---|
| Formaldehyde | 2.5 |
| Acetaldehyde | 1.6 |
| Propanal | 0.38 |
| 3-Methylbutanal | 0.04 |
| Acrolein | 0.11 |
| Methacrolein | 0.11 |
| Crotonaldehyde | 0.07 |
| Pentanal | 0.10 |
| Hexanal | 0.11 |
| Heptanal | 0.08 |
| Octanal | 0.10 |
| Nonanal | 0.60 |
| Decanal | 0.16 |
| 2-Nonenal | 0.05 |
| Acetone | 2.0 |
| 2-Butanone (MEK) | 0.22 |
| 3-Buten-2-one (MVK) | 0.11 |
| Cyclohexanone | 0.69 |
| Benzaldehyde | 0.06 |
| o-Tolualdehyde | 0.05 |

*continued on next page*

| **Species** | **Outdoor Mixing Ratio (ppb)** |
|---|---|
| m-Tolualdehyde | 0.08 |
| p-Tolualdehyde | 0.08 |
| 2,5-Dimethylbenzaldehyde | 0.32 |
| Benzene | 0.39 |
| Toluene | 1.7 |
| p-Xylene | 0.25 |
| m-Xylene | 0.25 |
| o-Xylene | 0.17 |
| Ethylbenzene | 0.36 |
| Propylbenzene | 0.16 |
| 2-Ethyltoluene | 0.01 |
| 3-Ethyltoluene | 0.02 |
| 4-Ethyltoluene | 0.01 |
| 1,3,5-Trimethylbenzene | 0.07 |
| 1,2,4-Trimethylbenzene | 0.22 |
| 1,2,3-Trimethylbenzene | 0.05 |
| Styrene | 0.09 |
| Cumene | 0.12 |
| Phenol | 0.71 |
| Ethane | 3.7 |
| Propane | 1.5 |
| Butane | 1.4 |
| Isobutane | 0.83 |
| 2,2-Dimethylbutane | 0.08 |

*continued on next page*

| **Species** | **Outdoor Mixing Ratio (ppb)** |
|---|---|
| 2,3-Dimethylbutane | 0.11 |
| Pentane | 0.35 |
| 2-Methylpentane | 0.16 |
| 3-Methylpentane | 0.10 |
| Isopentane | 0.60 |
| Hexane | 0.45 |
| 2-Methylhexane | 0.10 |
| 3-Methylhexane | 0.13 |
| Heptane | 0.02 |
| Octane | 0.02 |
| Nonane | 0.12 |
| Decane | 0.40 |
| Undecane | 0.59 |
| Dodecane | 0.04 |
| Cyclohexane | 0.03 |
| Ethene | 1.4 |
| Propene | 0.37 |
| 1-Butene | 0.16 |
| cis-2-Butene | 0.02 |
| trans-2-Butene | 0.02 |
| 2-Methyl-1-butene | 0.02 |
| 2-Methyl-2-butene | 0.02 |
| Isoprene | 0.09 |
| 1,3-Butadiene | 0.02 |

*continued on next page*

| **Species** | **Outdoor Mixing Ratio (ppb)** |
|---|---|
| trans-2-Pentene | 0.02 |
| cis-2-Pentene | 0.01 |
| Ethyne | 0.64 |
| Methanol | 4.5 |
| Ethanol | 6.6 |
| Isopropanol | 3.8 |
| 1-Propanol | 0.51 |
| 1-Butanol | 1.0 |
| 1-Pentanol | 0.002 |
| 1-Hexanol | 0.001 |
| 2-Butoxyethanol | 1.0 |
| Linalool | 0.001 |
| Chloroform | 0.03 |
| Methylchloroform | 0.31 |
| Dichloromethane | 0.10 |
| Trichloroethylene | 0.37 |
| Tetrachloroethylene | 0.02 |
| 1,2-Dichloroethane | 0.02 |
| Hydrogen Chloride | 1.5 |
| Chloromethane | 0.57 |
| Ethyl Acetate | 0.10 |
| Butyl Acetate | 0.05 |
| $\alpha$-Pinene | 0.13 |
| $\beta$-Pinene | 0.05 |

*continued on next page*

| **Species** | **Outdoor Mixing Ratio (ppb)** |
|---|---|
| Limonene | 0.10 |
| $\Delta$3-Carene | 0.11 |
| Camphene | 0.02 |
| Formic Acid | 7.5 |
| Acetic Acid | 15.7 |
| Propanoic Acid | 0.08 |
| Butanoic Acid | 0.06 |
| Pentanoic Acid | 0.03 |
| Heptanoic Acid | 0.004 |
| Hydrogen Peroxide | 1.3 |
| $\beta$-Caryophyllene | 0.004 |
| Methane ($CH_4$) | 1891 |
| Carbon Monoxide (CO) | 195 |
| Sulfur Dioxide ($SO_2$) | 0.70 |
| Nitric Acid ($HNO_3$) | 0.39 |
| Nitrous Acid (HONO) | 0.65 |
| Peroxyacetyl Nitrates (PAN) | 2.2 |

Table S5: The constant primary surface emission rates (in molecule $cm^{-3}$ $s^{-1}$) used in our simulated kitchen and classroom. The primary surface emissions are from wood and paint (Plaisance *et al.*, 2017; Alapieti *et al.*, 2021; Cheng *et al.*, 2015).

| **Species** | **Kitchen Emission Rate** | **Classroom Emission Rate** |
|---|---|---|
| Formaldehyde | $1.7 \times 10^{8}$ | $7.7 \times 10^{7}$ |
| Acetaldehyde | $9.6 \times 10^{7}$ | $5.2 \times 10^{7}$ |
| Propanal | $4.1 \times 10^{7}$ | $9.0 \times 10^{6}$ |
| Butanal | $5.0 \times 10^{7}$ | $1.1 \times 10^{7}$ |
| Pentanal | $4.1 \times 10^{7}$ | $1.6 \times 10^{7}$ |
| Hexanal | $1.1 \times 10^{8}$ | $4.4 \times 10^{7}$ |
| Heptanal | $5.2 \times 10^{6}$ | $2.9 \times 10^{6}$ |
| Octanal | $4.8 \times 10^{6}$ | $1.1 \times 10^{6}$ |
| Nonanal | $8.5 \times 10^{6}$ | $1.9 \times 10^{6}$ |
| Decanal | $4.5 \times 10^{6}$ | $9.9 \times 10^{5}$ |

Table S6: The photolysis rate coefficients ($s^{-1}$) for five far-UVC wavelength ranges (bins) at a distance of 20 cm from the 222 nm light source. The blank values indicate no absorption occurs.

| **Label** | **UV205** | **UV215** | **UV225** | **UV235** | **UV245** |
|---|---|---|---|---|---|
| J1 | 7.1 x $10^{-8}$ | 5.7 x $10^{-6}$ | 8.9 x $10^{-5}$ | 3.2 x $10^{-6}$ | 2.7 x $10^{-8}$ |
| J2 | 7.9 x $10^{-9}$ | 6.3 x $10^{-7}$ | 6.5 x $10^{-6}$ | 3.5 x $10^{-7}$ | 3.0 x $10^{-9}$ |
| J3 | 4.2 x $10^{-8}$ | 6.7 x $10^{-7}$ | 4.7 x $10^{-6}$ | 2.3 x $10^{-7}$ | 5.2 x $10^{-10}$ |
| J4 | 4.3 x $10^{-8}$ | 8.8 x $10^{-7}$ | 8.5 x $10^{-6}$ | 3.4 x $10^{-7}$ | 3.0 x $10^{-10}$ |
| J5 | - | - | - | - | - |
| J6 | - | - | - | - | - |
| J7 | 2.0 x $10^{-7}$ | 3.3 x $10^{-6}$ | 2.6 x $10^{-5}$ | 1.1 x $10^{-6}$ | 1.7 x $10^{-9}$ |
| J8 | 3.4 x $10^{-7}$ | 7.1 x $10^{-7}$ | 2.6 x $10^{-6}$ | 7.0 x $10^{-8}$ | 1.1 x $10^{-10}$ |
| J9 | 1.8 x $10^{-12}$ | 2.5 x $10^{-11}$ | 1.1 x $10^{-10}$ | 1.8 x $10^{-12}$ | 2.5 x $10^{-15}$ |
| J10 | 2.1 x $10^{-12}$ | 5.3 x $10^{-13}$ | 2.9 x $10^{-13}$ | 3.1 x $10^{-16}$ | - |
| J11 | - | - | 1.1 x $10^{-10}$ | 5.6 x $10^{-11}$ | 1.4 x $10^{-12}$ |
| J12 | - | - | 2.3 x $10^{-10}$ | 1.5 x $10^{-10}$ | 2.8 x $10^{-12}$ |
| J13 | 2.4 x $10^{-11}$ | 7.3 x $10^{-10}$ | 1.1 x $10^{-8}$ | 4.6 x $10^{-10}$ | 6.4 x $10^{-12}$ |
| J14 | 6.2 x $10^{-11}$ | 3.1 x $10^{-9}$ | 4.2 x $10^{-8}$ | 1.4 x $10^{-9}$ | 2.0 x $10^{-11}$ |
| J15 | 8.6 x $10^{-12}$ | 7.4 x $10^{-10}$ | 9.6 x $10^{-9}$ | 2.8 x $10^{-10}$ | 3.9 x $10^{-12}$ |
| J16 | 4.1 x $10^{-12}$ | 3.5 x $10^{-10}$ | 4.6 x $10^{-9}$ | 1.3 x $10^{-10}$ | 1.9 x $10^{-12}$ |
| J17 | 2.7 x $10^{-10}$ | 1.7 x $10^{-9}$ | 9.2 x $10^{-9}$ | 4.2 x $10^{-10}$ | 5.2 x $10^{-12}$ |
| J18 | - | - | - | - | - |
| J19 | - | - | - | - | - |
| J20 | - | - | - | - | - |
| J21 | - | 1.8 x $10^{-8}$ | 7.9 x $10^{-8}$ | 5.6 x $10^{-9}$ | 5.6 x $10^{-11}$ |

*continued on next page*

| Label | UV205 | UV215 | UV225 | UV235 | UV245 |
|---|---|---|---|---|---|
| J22 | $2.7 \times 10^{-10}$ | $3.5 \times 10^{-9}$ | $5.3 \times 10^{-8}$ | $1.8 \times 10^{-9}$ | $1.7 \times 10^{-11}$ |
| J23 | - | - | - | - | - |
| J24 | - | - | - | - | - |
| J31 | - | - | - | - | - |
| J32 | - | - | - | - | - |
| J33 | - | - | - | - | - |
| J34 | - | - | $4.6 \times 10^{-8}$ | $1.9 \times 10^{-8}$ | $9.6 \times 10^{-11}$ |
| J35 | $2.0 \times 10^{-8}$ | $1.2 \times 10^{-7}$ | $3.6 \times 10^{-7}$ | $9.5 \times 10^{-9}$ | $6.8 \times 10^{-11}$ |
| J41 | - | $4.0 \times 10^{-7}$ | $2.8 \times 10^{-6}$ | $1.2 \times 10^{-7}$ | $2.5 \times 10^{-10}$ |
| J51 | - | - | - | - | $2.4 \times 10^{-10}$ |
| J52 | $8.5 \times 10^{-7}$ | $3.9 \times 10^{-6}$ | $1.6 \times 10^{-5}$ | $3.0 \times 10^{-7}$ | $3.1 \times 10^{-10}$ |
| J53 | $9.6 \times 10^{-7}$ | $4.9 \times 10^{-6}$ | $1.8 \times 10^{-5}$ | $3.4 \times 10^{-7}$ | $3.5 \times 10^{-10}$ |
| J54 | $9.7 \times 10^{-7}$ | $5.0 \times 10^{-6}$ | $2.2 \times 10^{-5}$ | $4.2 \times 10^{-7}$ | $4.3 \times 10^{-10}$ |
| J55 | $9.9 \times 10^{-7}$ | $5.3 \times 10^{-6}$ | $2.0 \times 10^{-5}$ | $3.7 \times 10^{-7}$ | $3.9 \times 10^{-10}$ |
| J56 | - | - | - | - | $6.3 \times 10^{-10}$ |
| J57 | - | - | - | - | $6.3 \times 10^{-10}$ |
| J70 | $9.3 \times 10^{-12}$ | $2.5 \times 10^{-9}$ | $1.4 \times 10^{-8}$ | $6.7 \times 10^{-10}$ | $2.4 \times 10^{-12}$ |
| J71 | $3.9 \times 10^{-7}$ | $6.3 \times 10^{-6}$ | $6.0 \times 10^{-5}$ | $2.8 \times 10^{-6}$ | $5.7 \times 10^{-9}$ |
| J72 | $1.6 \times 10^{-7}$ | $3.7 \times 10^{-6}$ | $3.6 \times 10^{-5}$ | $1.6 \times 10^{-6}$ | $2.6 \times 10^{-9}$ |
| J73 | $1.1 \times 10^{-7}$ | $2.5 \times 10^{-6}$ | $2.4 \times 10^{-5}$ | $1.0 \times 10^{-6}$ | $1.7 \times 10^{-9}$ |
| J74 | $9.2 \times 10^{-9}$ | $3.2 \times 10^{-7}$ | $3.5 \times 10^{-6}$ | $1.7 \times 10^{-7}$ | $6.9 \times 10^{-10}$ |
| J75 | - | - | - | - | - |
| J76 | - | - | - | - | - |

*continued on next page*

| **Label** | **UV205** | **UV215** | **UV225** | **UV235** | **UV245** |
|---|---|---|---|---|---|
| J77 | - | - | - | - | - |
| J78 | - | - | - | - | - |

Table S7: The photolysis rate coefficients ($s^{-1}$) for five near-UVC wavelength ranges (bins) at a distance of 20 cm from the 222 nm light source. The blank values indicate no absorption occurs.

| **Label** | **UV255** | **UV265** | **UV275** | **UV285** | **UV295** |
|---|---|---|---|---|---|
| J1 | $8.2 \times 10^{-8}$ | $1.3 \times 10^{-8}$ | $1.5 \times 10^{-8}$ | $1.8 \times 10^{-8}$ | $5.7 \times 10^{-9}$ |
| J2 | $9.1 \times 10^{-9}$ | $1.4 \times 10^{-9}$ | $1.7 \times 10^{-9}$ | $2.0 \times 10^{-9}$ | $6.4 \times 10^{-10}$ |
| J3 | $3.7 \times 10^{-10}$ | $1.5 \times 10^{-10}$ | $5.8 \times 10^{-11}$ | $1.1 \times 10^{-10}$ | $9.7 \times 10^{-11}$ |
| J4 | $6.1 \times 10^{-11}$ | $4.7 \times 10^{-11}$ | $8.2 \times 10^{-11}$ | $3.9 \times 10^{-10}$ | $8.6 \times 10^{-10}$ |
| J5 | - | - | - | - | - |
| J6 | - | - | - | - | - |
| J7 | $7.2 \times 10^{-10}$ | $2.2 \times 10^{-10}$ | $5.7 \times 10^{-11}$ | - | - |
| J8 | $9.8 \times 10^{-11}$ | $5.3 \times 10^{-11}$ | $3.0 \times 10^{-11}$ | $6.1 \times 10^{-11}$ | $4.6 \times 10^{-11}$ |
| J9 | - | - | - | - | - |
| J10 | - | - | - | - | - |
| J11 | $1.1 \times 10^{-11}$ | $4.1 \times 10^{-12}$ | $2.6 \times 10^{-11}$ | $1.1 \times 10^{-10}$ | $1.7 \times 10^{-10}$ |
| J12 | $1.7 \times 10^{-11}$ | $5.2 \times 10^{-12}$ | $2.0 \times 10^{-11}$ | $5.1 \times 10^{-11}$ | $6.7 \times 10^{-11}$ |
| J13 | $4.3 \times 10^{-11}$ | $1.2 \times 10^{-11}$ | $6.0 \times 10^{-11}$ | $2.0 \times 10^{-10}$ | $1.9 \times 10^{-10}$ |
| J14 | $1.4 \times 10^{-10}$ | $3.4 \times 10^{-11}$ | $1.2 \times 10^{-10}$ | $3.7 \times 10^{-10}$ | $4.7 \times 10^{-10}$ |
| J15 | $2.9 \times 10^{-11}$ | $7.5 \times 10^{-12}$ | $2.9 \times 10^{-11}$ | $8.6 \times 10^{-11}$ | $1.0 \times 10^{-10}$ |
| J16 | $1.4 \times 10^{-11}$ | $3.6 \times 10^{-12}$ | $1.4 \times 10^{-11}$ | $4.1 \times 10^{-11}$ | $4.9 \times 10^{-11}$ |
| J17 | $2.6 \times 10^{-11}$ | $1.1 \times 10^{-11}$ | $4.5 \times 10^{-11}$ | $2.6 \times 10^{-10}$ | $4.5 \times 10^{-10}$ |
| J18 | $7.4 \times 10^{-13}$ | $1.9 \times 10^{-13}$ | $1.2 \times 10^{-12}$ | $5.4 \times 10^{-12}$ | $1.1 \times 10^{-11}$ |
| J19 | $7.4 \times 10^{-13}$ | $1.9 \times 10^{-13}$ | $1.2 \times 10^{-12}$ | $5.4 \times 10^{-12}$ | $1.1 \times 10^{-11}$ |
| J20 | $7.4 \times 10^{-13}$ | $1.9 \times 10^{-13}$ | $1.2 \times 10^{-12}$ | $5.4 \times 10^{-12}$ | $1.1 \times 10^{-11}$ |
| J21 | $2.6 \times 10^{-10}$ | $6.3 \times 10^{-11}$ | $1.5 \times 10^{-10}$ | $3.3 \times 10^{-10}$ | $3.0 \times 10^{-10}$ |

*continued on next page*

| Label | UV255 | UV265 | UV275 | UV285 | UV295 |
|---|---|---|---|---|---|
| J22 | 9.4 x $10^{-11}$ | 2.1 x $10^{-11}$ | 5.6 x $10^{-11}$ | 1.3 x $10^{-10}$ | 1.2 x $10^{-10}$ |
| J23 | 7.4 x $10^{-12}$ | 1.2 x $10^{-12}$ | 3.5 x $10^{-12}$ | 1.5 x $10^{-11}$ | 1.0 x $10^{-11}$ |
| J24 | 7.4 x $10^{-12}$ | 1.2 x $10^{-12}$ | 3.5 x $10^{-12}$ | 1.5 x $10^{-11}$ | 1.0 x $10^{-11}$ |
| J31 | 6.6 x $10^{-11}$ | 1.3 x $10^{-11}$ | 2.8 x $10^{-11}$ | 4.9 x $10^{-11}$ | 3.2 x $10^{-11}$ |
| J32 | 1.7 x $10^{-11}$ | 2.2 x $10^{-12}$ | 2.8 x $10^{-11}$ | 1.0 x $10^{-10}$ | 1.5 x $10^{-10}$ |
| J33 | 6.9 x $10^{-13}$ | 3.2 x $10^{-13}$ | 1.9 x $10^{-12}$ | 1.0 x $10^{-11}$ | 2.5 x $10^{-11}$ |
| J34 | 1.4 x $10^{-10}$ | 9.5 x $10^{-11}$ | 9.3 x $10^{-11}$ | 3.1 x $10^{-10}$ | 4.2 x $10^{-10}$ |
| J35 | 2.3 x $10^{-10}$ | 5.2 x $10^{-11}$ | 1.1 x $10^{-10}$ | 2.3 x $10^{-10}$ | 1.8 x $10^{-10}$ |
| J41 | 1.8 x $10^{-10}$ | 7.2 x $10^{-11}$ | 3.1 x $10^{-11}$ | 6.2 x $10^{-11}$ | 5.7 x $10^{-11}$ |
| J51 | 1.7 x $10^{-10}$ | 8.7 x $10^{-11}$ | 4.4 x $10^{-11}$ | 8.7 x $10^{-11}$ | 6.6 x $10^{-11}$ |
| J52 | 2.7 x $10^{-10}$ | 1.1 x $10^{-10}$ | 5.9 x $10^{-11}$ | 1.2 x $10^{-10}$ | 9.5 x $10^{-11}$ |
| J53 | 2.3 x $10^{-10}$ | 1.1 x $10^{-10}$ | 6.1 x $10^{-11}$ | 1.3 x $10^{-10}$ | 1.0 x $10^{-10}$ |
| J54 | 2.6 x $10^{-10}$ | 1.3 x $10^{-10}$ | 7.5 x $10^{-11}$ | 1.6 x $10^{-10}$ | 1.3 x $10^{-10}$ |
| J55 | 2.5 x $10^{-10}$ | 1.2 x $10^{-10}$ | 6.6 x $10^{-11}$ | 1.4 x $10^{-10}$ | 1.1 x $10^{-10}$ |
| J56 | 5.5 x $10^{-10}$ | 2.2 x $10^{-10}$ | 1.4 x $10^{-10}$ | 3.7 x $10^{-10}$ | 4.5 x $10^{-10}$ |
| J57 | 5.5 x $10^{-10}$ | 2.2 x $10^{-10}$ | 1.4 x $10^{-10}$ | 3.7 x $10^{-10}$ | 4.5 x $10^{-10}$ |
| J70 | 1.1 x $10^{-12}$ | 1.9 x $10^{-11}$ | 7.3 x $10^{-12}$ | 1.4 x $10^{-10}$ | 6.2 x $10^{-10}$ |
| J71 | 4.2 x $10^{-9}$ | 1.7 x $10^{-9}$ | 1.1 x $10^{-10}$ | 1.3 x $10^{-9}$ | 1.5 x $10^{-9}$ |
| J72 | 1.4 x $10^{-9}$ | 5.7 x $10^{-10}$ | 2.1 x $10^{-10}$ | 3.2 x $10^{-10}$ | 3.0 x $10^{-10}$ |
| J73 | 9.6 x $10^{-10}$ | 3.8 x $10^{-10}$ | 1.4 x $10^{-10}$ | 2.6 x $10^{-10}$ | 2.0 x $10^{-10}$ |
| J74 | 1.1 x $10^{-9}$ | 1.8 x $10^{-10}$ | 1.5 x $10^{-10}$ | 3.4 x $10^{-10}$ | 4.7 x $10^{-10}$ |
| J75 | - | - | 1.3 x $10^{-9}$ | 5.3 x $10^{-9}$ | 1.2 x $10^{-8}$ |
| J76 | - | - | - | - | - |

*continued on next page*

| Label | UV255 | UV265 | UV275 | UV285 | UV295 |
|---|---|---|---|---|---|
| J77 | - | - | - | - | - |
| J78 | - | - | - | - | - |

Table S8: The photolysis rate coefficients ($s^{-1}$) in a classroom with GUV222 lamps with respective average room irradiances. The blank values indicate no absorption occurs.

| **Label** | **GUV222** **1 $\mu$W cm$^{-2}$** | **GUV222** **3 $\mu$W cm$^{-2}$** | **GUV222** **5 $\mu$W cm$^{-2}$** |
|---|---|---|---|
| J1 | 2.3 x $10^{-6}$ | 7.0 x $10^{-6}$ | 1.1 x $10^{-5}$ |
| J2 | 2.5 x $10^{-7}$ | 7.7 x $10^{-7}$ | 1.3 x $10^{-6}$ |
| J3 | 2.8 x $10^{-7}$ | 8.8 x $10^{-7}$ | 1.4 x $10^{-6}$ |
| J4 | 5.1 x $10^{-7}$ | 1.6 x $10^{-6}$ | 2.6 x $10^{-6}$ |
| J5 | - | - | - |
| J6 | - | - | - |
| J7 | 1.6 x $10^{-6}$ | 5.0 x $10^{-6}$ | 8.2 x $10^{-6}$ |
| J8 | 1.5 x $10^{-7}$ | 4.6 x $10^{-7}$ | 7.5 x $10^{-7}$ |
| J9 | 4.5 x $10^{-12}$ | 1.4 x $10^{-11}$ | 2.3 x $10^{-11}$ |
| J10 | 1.0 x $10^{-14}$ | 3.2 x $10^{-14}$ | 5.2 x $10^{-14}$ |
| J11 | 5.4 x $10^{-11}$ | 1.7 x $10^{-10}$ | 2.7 x $10^{-10}$ |
| J12 | 1.5 x $10^{-10}$ | 4.5 x $10^{-10}$ | 7.4 x $10^{-10}$ |
| J13 | 2.2 x $10^{-10}$ | 6.6 x $10^{-10}$ | 1.1 x $10^{-9}$ |
| J14 | 8.5 x $10^{-10}$ | 2.6 x $10^{-9}$ | 4.3 x $10^{-9}$ |
| J15 | 2.0 x $10^{-10}$ | 6.1 x $10^{-10}$ | 1.0 x $10^{-9}$ |
| J16 | 9.4 x $10^{-11}$ | 2.9 x $10^{-10}$ | 4.8 x $10^{-10}$ |
| J17 | 3.1 x $10^{-10}$ | 9.5 x $10^{-10}$ | 1.6 x $10^{-9}$ |
| J18 | - | - | - |
| J19 | - | - | - |
| J20 | - | - | - |
| J21 | 3.2 x $10^{-9}$ | 1.0 x $10^{-8}$ | 1.7 x $10^{-8}$ |
| J22 | 1.1 x $10^{-9}$ | 3.4 x $10^{-9}$ | 5.5 x $10^{-9}$ |

*continued on next page*

| **Label** | **GUV222 1 $\mu$W cm$^{-2}$** | **GUV222 3 $\mu$W cm$^{-2}$** | **GUV222 5 $\mu$W cm$^{-2}$** |
|---|---|---|---|
| J23 | - | - | - |
| J24 | - | - | - |
| J31 | - | - | - |
| J32 | - | - | - |
| J33 | - | - | - |
| J34 | 1.4 x $10^{-8}$ | 4.3 x $10^{-8}$ | 7.1 x $10^{-8}$ |
| J35 | 1.4 x $10^{-8}$ | 4.4 x $10^{-8}$ | 7.2 x $10^{-8}$ |
| J41 | 1.7 x $10^{-7}$ | 5.2 x $10^{-7}$ | 8.6 x $10^{-7}$ |
| J51 | - | - | - |
| J52 | 1.0 x $10^{-6}$ | 3.1 x $10^{-6}$ | 5.1 x $10^{-6}$ |
| J53 | 1.2 x $10^{-6}$ | 3.6 x $10^{-6}$ | 5.9 x $10^{-6}$ |
| J54 | 1.4 x $10^{-6}$ | 4.3 x $10^{-6}$ | 7.0 x $10^{-6}$ |
| J55 | 1.2 x $10^{-6}$ | 3.8 x $10^{-6}$ | 6.2 x $10^{-6}$ |
| J56 | - | - | - |
| J57 | - | - | - |
| J70 | 5.6 x $10^{-10}$ | 1.7 x $10^{-9}$ | 2.8 x $10^{-9}$ |
| J71 | 3.6 x $10^{-6}$ | 1.1 x $10^{-5}$ | 1.8 x $10^{-5}$ |
| J72 | 2.2 x $10^{-6}$ | 6.8 x $10^{-6}$ | 1.1 x $10^{-5}$ |
| J73 | 1.5 x $10^{-6}$ | 4.5 x $10^{-6}$ | 7.4 x $10^{-6}$ |
| J74 | 1.3 x $10^{-7}$ | 3.9 x $10^{-7}$ | 6.5 x $10^{-7}$ |
| J75 | - | - | - |
| J76 | - | - | - |
| J77 | - | - | - |

*continued on next page*

| **Label** | **GUV222 1 $\mu$W cm$^{-2}$** | **GUV222 3 $\mu$W cm$^{-2}$** | **GUV222 5 $\mu$W cm$^{-2}$** |
|---|---|---|---|
| J78 | - | - | - |

Table S9: The photolysis rate coefficients ($s^{-1}$) in a classroom with GUV254 lamps with respective average room irradiances. The blank values indicate no absorption occurs.

| **Label** | **GUV254** **9 $\mu$W cm$^{-2}$** | **GUV254** **12 $\mu$W cm$^{-2}$** | **GUV254** **15 $\mu$W cm$^{-2}$** |
|---|---|---|---|
| J1 | 1.3 x $10^{-4}$ | 1.5 x $10^{-4}$ | 2.0 x $10^{-4}$ |
| J2 | 1.3 x $10^{-5}$ | 1.7 x $10^{-5}$ | 2.2 x $10^{-5}$ |
| J3 | 7.4 x $10^{-7}$ | 1.0 x $10^{-6}$ | 1.3 x $10^{-6}$ |
| J4 | 1.2 x $10^{-7}$ | 1.6 x $10^{-7}$ | 2.1 x $10^{-7}$ |
| J5 | - | - | - |
| J6 | - | - | - |
| J7 | 1.4 x $10^{-6}$ | 1.9 x $10^{-6}$ | 2.4 x $10^{-6}$ |
| J8 | 2.1 x $10^{-7}$ | 2.8 x $10^{-7}$ | 3.6 x $10^{-7}$ |
| J9 | - | - | - |
| J10 | - | - | - |
| J11 | 1.2 x $10^{-8}$ | 1.6 x $10^{-8}$ | 2.1 x $10^{-8}$ |
| J12 | 1.8 x $10^{-8}$ | 2.5 x $10^{-8}$ | 3.1 x $10^{-8}$ |
| J13 | 5.0 x $10^{-8}$ | 6.9 x $10^{-8}$ | 8.7 x $10^{-8}$ |
| J14 | 1.6 x $10^{-7}$ | 2.2 x $10^{-7}$ | 2.8 x $10^{-7}$ |
| J15 | 3.4 x $10^{-8}$ | 4.6 x $10^{-8}$ | 5.8 x $10^{-8}$ |
| J16 | 1.6 x $10^{-8}$ | 2.2 x $10^{-8}$ | 2.8 x $10^{-8}$ |
| J17 | 4.3 x $10^{-8}$ | 5.9 x $10^{-8}$ | 7.5 x $10^{-8}$ |
| J18 | 3.8 x $10^{-11}$ | 5.2 x $10^{-11}$ | 6.6 x $10^{-11}$ |
| J19 | 3.8 x $10^{-11}$ | 5.2 x $10^{-11}$ | 6.6 x $10^{-11}$ |
| J20 | 2.0 x $10^{-8}$ | 2.7 x $10^{-8}$ | 3.4 x $10^{-8}$ |
| J21 | 3.3 x $10^{-7}$ | 4.5 x $10^{-7}$ | 5.7 x $10^{-7}$ |
| J22 | 1.2 x $10^{-7}$ | 1.6 x $10^{-7}$ | 2.0 x $10^{-7}$ |

*continued on next page*

| Label | **GUV254** **9 $\mu$W cm$^{-2}$** | **GUV254** **12 $\mu$W cm$^{-2}$** | **GUV254** **15 $\mu$W cm$^{-2}$** |
|---|---|---|---|
| J23 | 9.3 x $10^{-9}$ | 1.3 x $10^{-8}$ | 1.6 x $10^{-8}$ |
| J24 | 9.3 x $10^{-9}$ | 1.3 x $10^{-8}$ | 1.6 x $10^{-8}$ |
| J31 | 6.9 x $10^{-8}$ | 9.5 x $10^{-8}$ | 1.2 x $10^{-7}$ |
| J32 | 5.9 x $10^{-8}$ | 8.0 x $10^{-8}$ | 1.0 x $10^{-7}$ |
| J33 | 4.8 x $10^{-8}$ | 6.5 x $10^{-8}$ | 8.2 x $10^{-8}$ |
| J34 | 3.1 x $10^{-7}$ | 4.3 x $10^{-7}$ | 5.4 x $10^{-7}$ |
| J35 | 3.1 x $10^{-7}$ | 4.3 x $10^{-7}$ | 5.4 x $10^{-7}$ |
| J41 | 3.6 x $10^{-7}$ | 4.9 x $10^{-7}$ | 6.1 x $10^{-7}$ |
| J51 | 3.6 x $10^{-7}$ | 5.0 x $10^{-7}$ | 6.3 x $10^{-7}$ |
| J52 | 4.5 x $10^{-7}$ | 6.2 x $10^{-7}$ | 7.8 x $10^{-7}$ |
| J53 | 4.8 x $10^{-7}$ | 6.6 x $10^{-7}$ | 8.4 x $10^{-7}$ |
| J54 | 5.4 x $10^{-7}$ | 7.4 x $10^{-7}$ | 9.3 x $10^{-7}$ |
| J55 | 5.1 x $10^{-7}$ | 6.9 x $10^{-7}$ | 8.7 x $10^{-7}$ |
| J56 | 1.1 x $10^{-6}$ | 1.4 x $10^{-6}$ | 1.8 x $10^{-6}$ |
| J57 | 1.1 x $10^{-6}$ | 1.4 x $10^{-6}$ | 1.8 x $10^{-6}$ |
| J70 | 4.4 x $10^{-9}$ | 6.0 x $10^{-9}$ | 7.6 x $10^{-9}$ |
| J71 | 8.4 x $10^{-6}$ | 1.1 x $10^{-5}$ | 1.5 x $10^{-5}$ |
| J72 | 2.9 x $10^{-6}$ | 3.9 x $10^{-6}$ | 4.9 x $10^{-6}$ |
| J73 | 1.9 x $10^{-6}$ | 2.6 x $10^{-6}$ | 3.3 x $10^{-6}$ |
| J74 | 1.6 x $10^{-6}$ | 2.2 x $10^{-6}$ | 2.8 x $10^{-6}$ |
| J75 | - | - | - |
| J76 | - | - | - |
| J77 | - | - | - |

*continued on next page*

| Label | GUV254 9 $\mu$W cm$^{-2}$ | GUV254 12 $\mu$W cm$^{-2}$ | GUV254 15 $\mu$W cm$^{-2}$ |
|---|---|---|---|
| J78 | - | - | - |

Table S10: The emission rates from breath (in molecule $cm^{-3}$ $s^{-1}$) in the simulated classroom. These rates are based on Kruza and Carslaw (2019) and adapted based on the volume of the classroom and for twenty children and one adult.

| **Species** | **Emission Rate** |
|---|---|
| Acetone | $1.3 \times 10^8$ |
| Ethanol | $8.4 \times 10^7$ |
| Methanol | $7.4 \times 10^7$ |
| Isopropanol | $1.8 \times 10^7$ |
| Isoprene | $1.8 \times 10^7$ |

Table S11: The average indoor concentration for a range of key species (07:00 to 19:00 h) in a simulated kitchen at an ACR of 0.5 $h^{-1}$ for a selection of different lighting conditions. The units are molecule $cm^{-3}$ for the concentration of OH, ppt for the concentrations of $HO_2$, $RO_2$ and organic $NO_3$ ($RNO_3$) and ppb for the other species.

| | Concentration | | | | | | | | | |
|---|---|---|---|---|---|---|---|---|---|---|
| Label | OH | $O_3$ | $HO_2$ | $RO_2$ | NO | $NO_2$ | HONO | HCHO | PANs | $RNO_3$ |
| Dark | $4.6 \times 10^4$ | 2.6 | 1.4 | 2.5 | 0.3 | 0.9 | 0.07 | 7.2 | 0.5 | 8.1 |
| Incand | $4.8 \times 10^4$ | 2.6 | 1.5 | 2.5 | 0.3 | 0.9 | 0.07 | 7.2 | 0.5 | 8.4 |
| UV205 | $6.7 \times 10^4$ | 3.0 | 2.0 | 3.6 | 0.2 | 0.9 | 0.07 | 7.3 | 0.5 | 10.3 |
| UV215 | $3.6 \times 10^4$ | 9.5 | 2.0 | 4.6 | 0.1 | 0.9 | 0.07 | 7.8 | 0.5 | 5.6 |
| UV225 | $2.4 \times 10^5$ | 33.0 | 5.1 | 41.3 | 0.03 | 0.6 | 0.05 | 9.7 | 1.0 | 17.5 |
| UV235 | $4.3 \times 10^4$ | 3.0 | 1.5 | 2.6 | 0.3 | 0.9 | 0.07 | 7.3 | 0.5 | 7.5 |
| UV245 | $4.6 \times 10^4$ | 2.5 | 1.4 | 2.5 | 0.3 | 0.9 | 0.07 | 7.2 | 0.5 | 8.1 |
| UV255 | $4.6 \times 10^4$ | 2.5 | 1.4 | 2.5 | 0.3 | 0.9 | 0.07 | 7.2 | 0.5 | 8.1 |
| UV265 | $4.6 \times 10^4$ | 2.5 | 1.4 | 2.5 | 0.3 | 0.9 | 0.07 | 7.2 | 0.5 | 8.1 |
| UV275 | $4.6 \times 10^4$ | 2.5 | 1.4 | 2.5 | 0.3 | 0.9 | 0.07 | 7.2 | 0.5 | 8.1 |
| UV285 | $4.6 \times 10^4$ | 2.5 | 1.4 | 2.5 | 0.3 | 0.9 | 0.07 | 7.2 | 0.5 | 8.1 |
| UV295 | $4.6 \times 10^4$ | 2.5 | 1.4 | 2.5 | 0.3 | 0.9 | 0.07 | 7.2 | 0.5 | 8.1 |

Table S12: The net loss rate of ozone ($h^{-1}$) through chemistry ($O_3$ $LR_{Chemistry}$) and ventilation ($O_3$ $LR_{Ventilation}$). These loss rates add up to the total loss rate of ozone ($O_3$ TLR) given in Table 2 in the main paper.

| Simulation Number | $O_3$ $LR_{Chemistry}$ ($h^{-1}$) | $O_3$ $LR_{Ventilation}$ ($h^{-1}$) |
|---|---|---|
| 1 | 4.18 | 0.13 |
| 2 | 4.07 | 0.13 |
| 3 | 4.03 | 0.13 |
| 4 | 4.60 | 0.13 |
| 5 | 4.67 | 0.13 |
| 6 | 4.81 | 0.13 |
| 7 | 4.47 | 0.50 |
| 8 | 4.34 | 0.50 |
| 9 | 4.27 | 0.50 |
| 10 | 4.84 | 0.50 |
| 11 | 4.90 | 0.50 |
| 12 | 5.03 | 0.50 |
| 13 | 4.65 | 2.00 |
| 14 | 4.59 | 2.00 |
| 15 | 4.54 | 2.00 |
| 16 | 5.03 | 2.00 |
| 17 | 5.10 | 2.00 |
| 18 | 5.22 | 2.00 |
| 19 | 1.28 | 0.13 |
| 20 | 1.41 | 0.50 |
| 21 | 1.64 | 2.00 |

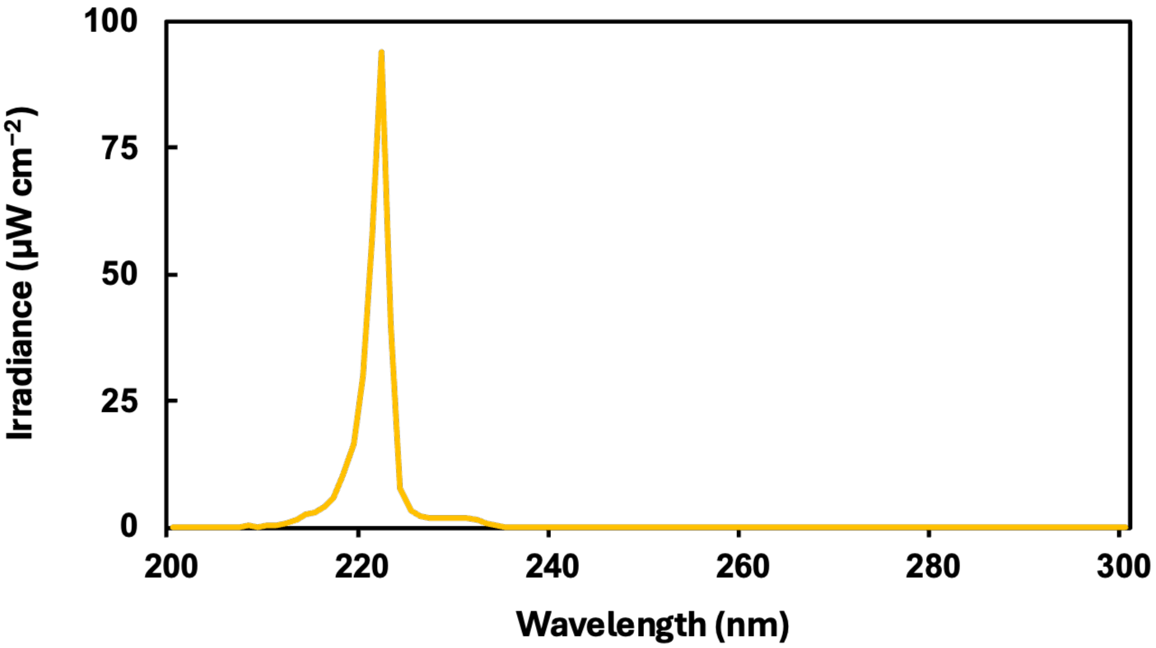


Figure S1: The measured spectral irradiance 20 cm from a KrCl excimer lamp focussed at 222 nm (Eadie *et al.*, 2022).

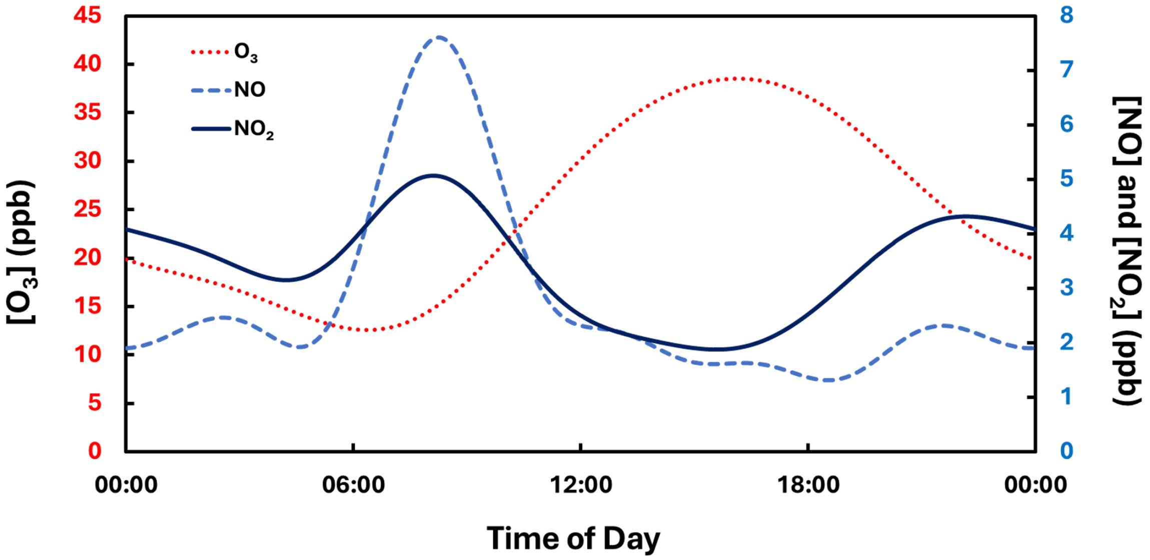


Figure S2: The measured diurnal outdoor mixing ratio profile of ozone, nitrogen oxide and nitrogen dioxide used in the classroom and kitchen simulations Shaw *et al.* (2023). The red dotted line is the outdoor mixing ratio of ozone, which follows the left-hand side axis. The lighter blue dashed line is the outdoor mixing ratio of nitrogen oxide, which follows the right-hand side axis. The darker blue solid line is the outdoor mixing ratio of nitrogen dioxide, which also follows the right-hand side axis.

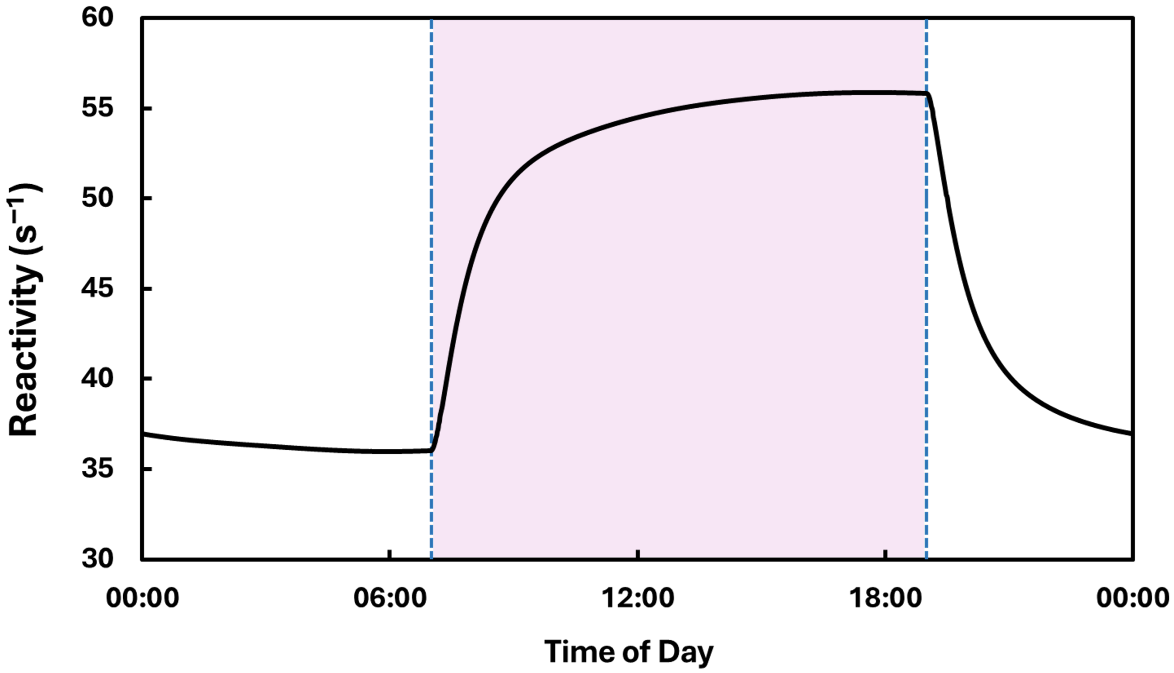


Figure S3: The reactivity ($s^{-1}$) of OH during operation of the UV225 light. The blue dashed lines and purple shaded area indicate the lights turning on at 07:00 h and off again at 19:00 h.

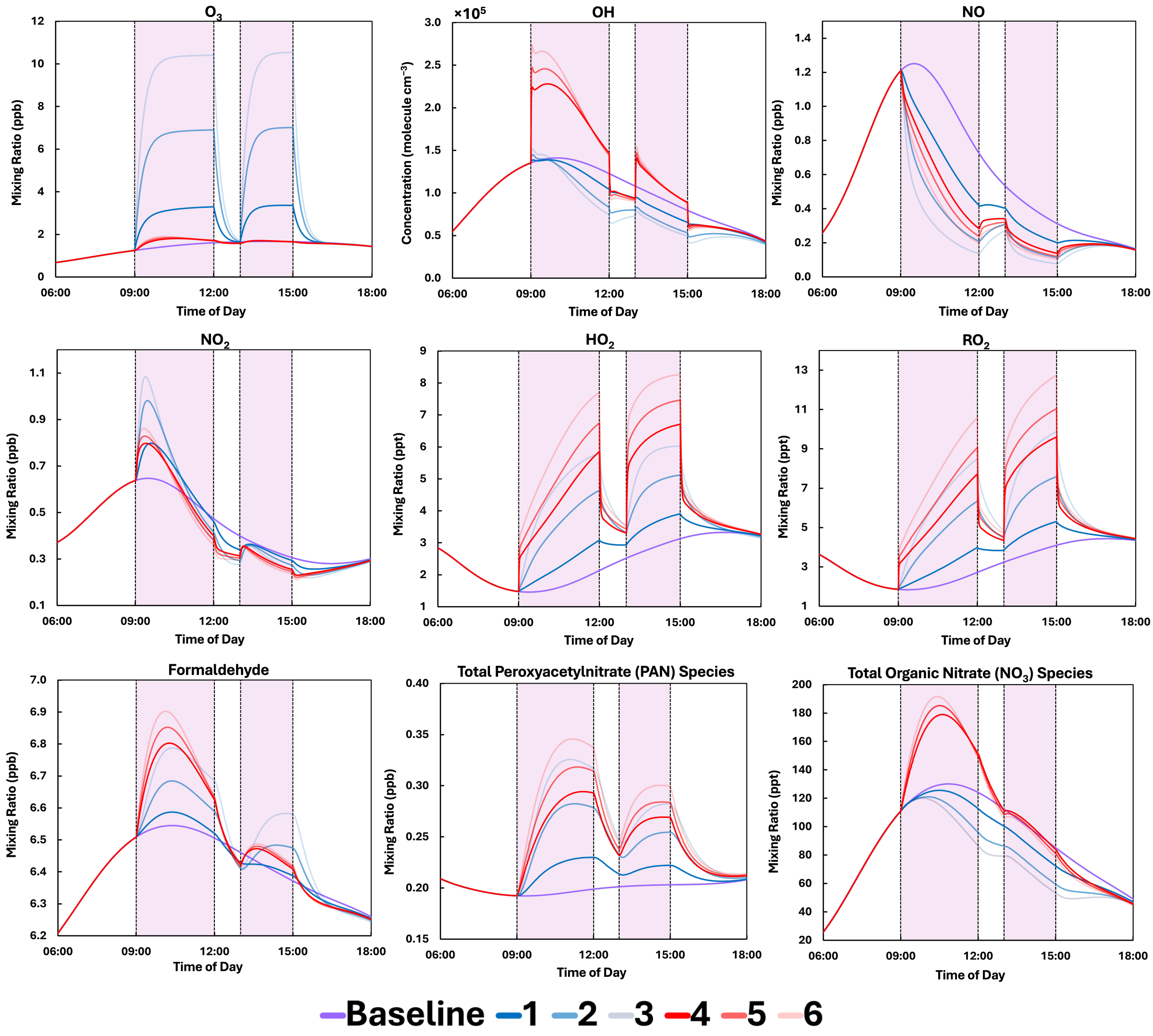


Figure S4: Diurnal concentrations in the simulated classroom at an ACR of 0.125 $h^{-1}$ with GUV222 or GUV254 lamps, one teacher and 20 students. Simulations 1-3 use a GUV222 lamp with an average room irradiance of 1, 3 and 5 $\mu$W $cm^{-2}$ respectively. Simulations 4-6 use a GUV254 lamp with an average room irradiance of 9, 12 and 15 $\mu$W $cm^{-2}$ respectively. The baseline simulation had no lamp present, and all simulations had attenuated outdoor lighting. The lamps are on from 09:00-12:00 h, and from 13:00-15:00 h, as indicated by the purple areas.

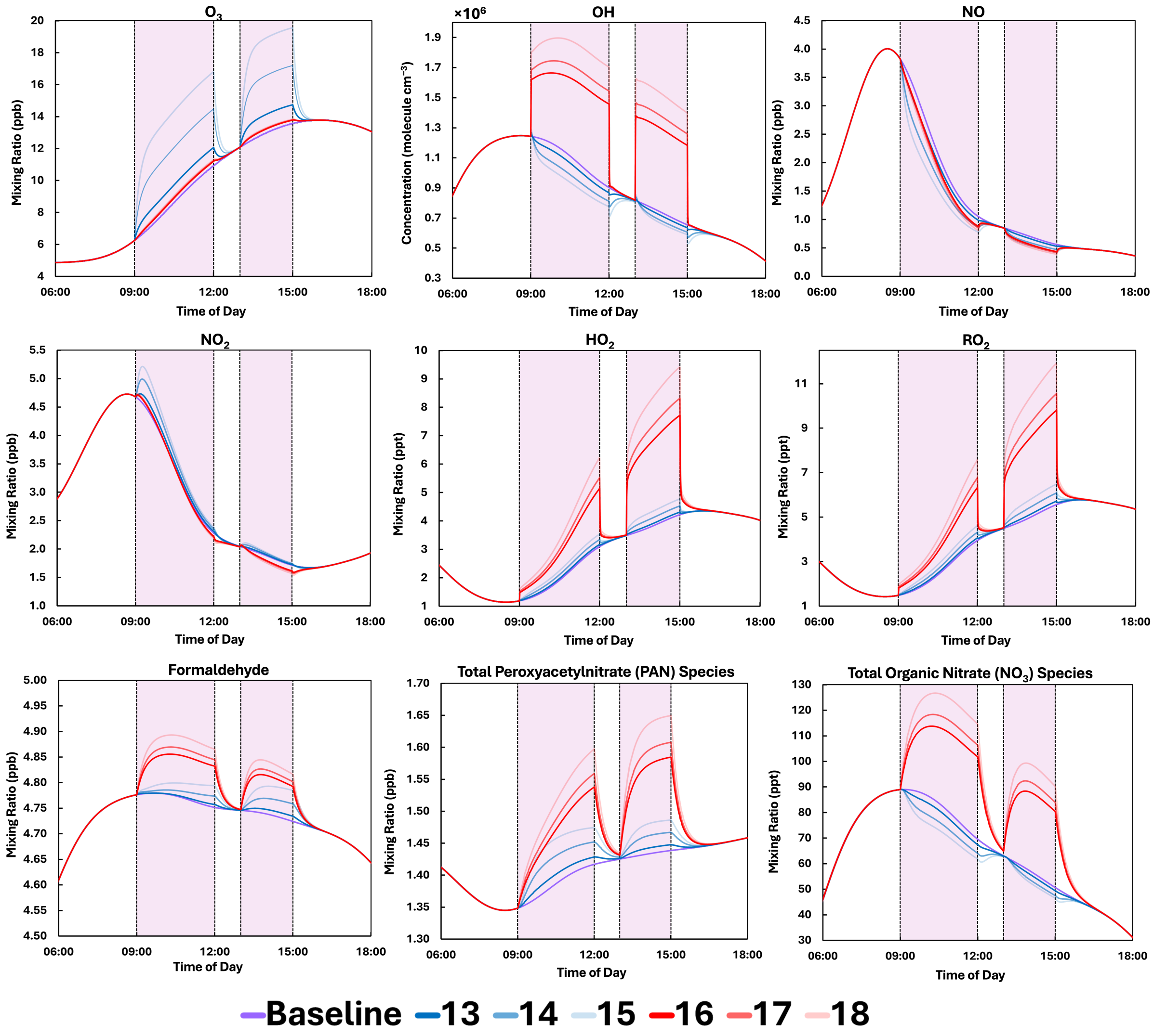


Figure S5: Diurnal concentrations in the simulated classroom at an ACR of 2.0 $h^{-1}$ with GUV222 or GUV254 lamps, one teacher and 20 students. Simulations 13-15 use a GUV222 lamp with an average room irradiance of 1, 3 and 5 $\mu W\ cm^{-2}$ respectively. Simulations 16-18 use a GUV254 lamp with an average room irradiance of 9, 12 and 15 $\mu W\ cm^{-2}$ respectively. The baseline simulation had no lamp present, and all simulations had attenuated outdoor lighting. The lamps are on from 09:00-12:00 h, and from 13:00-15:00 h, as indicated by the purple areas.

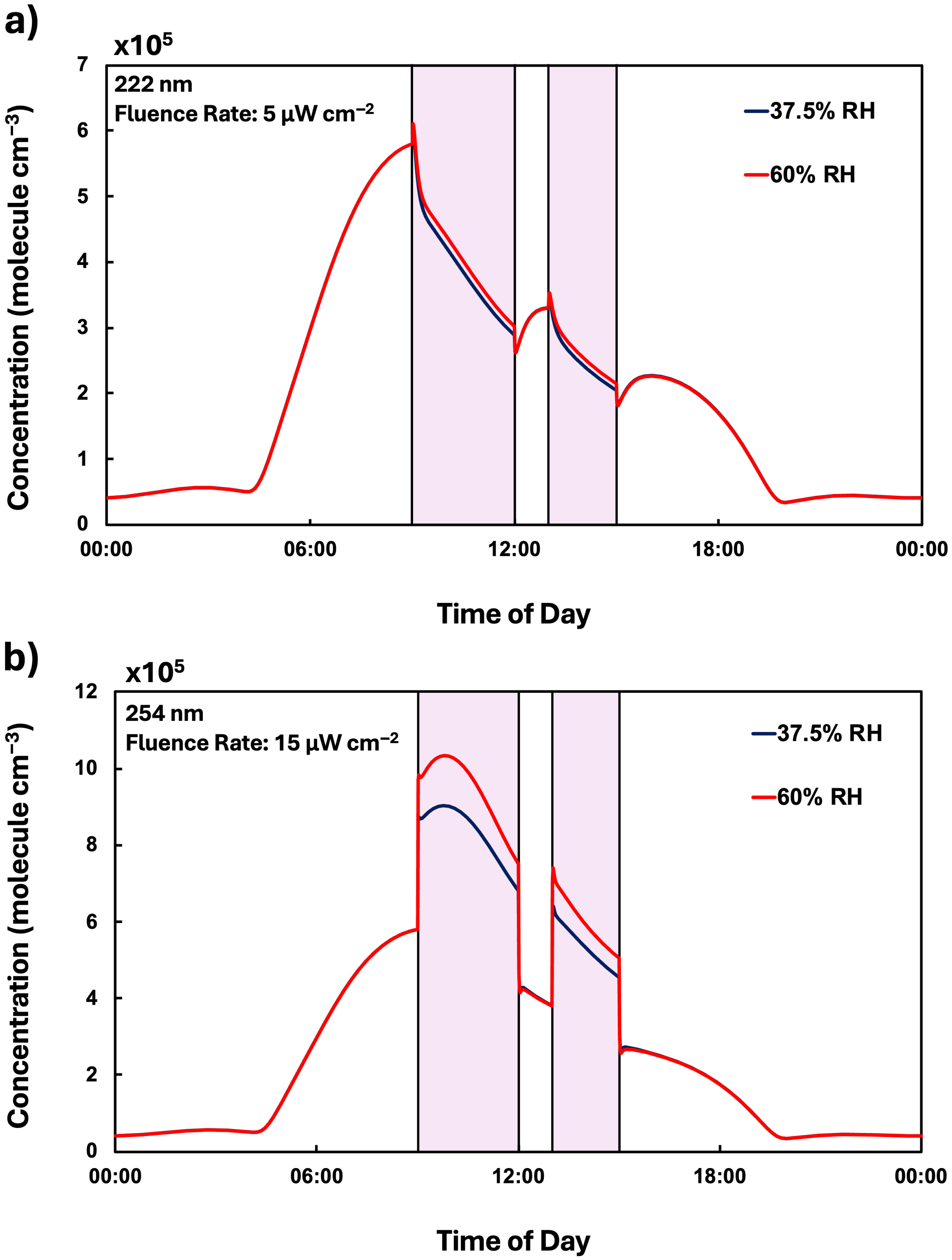


Figure S6: The concentration of OH following variation in relative humidity (%) with the addition of a GUV222 5 $\mu$W cm$^{-2}$ lamp and a GUV254 50 $\mu$W cm$^{-2}$ lamp (a) and b) respectively). The blue line in a) corresponds to Simulation 9 and the blue line in b) corresponds to Simulation 12.

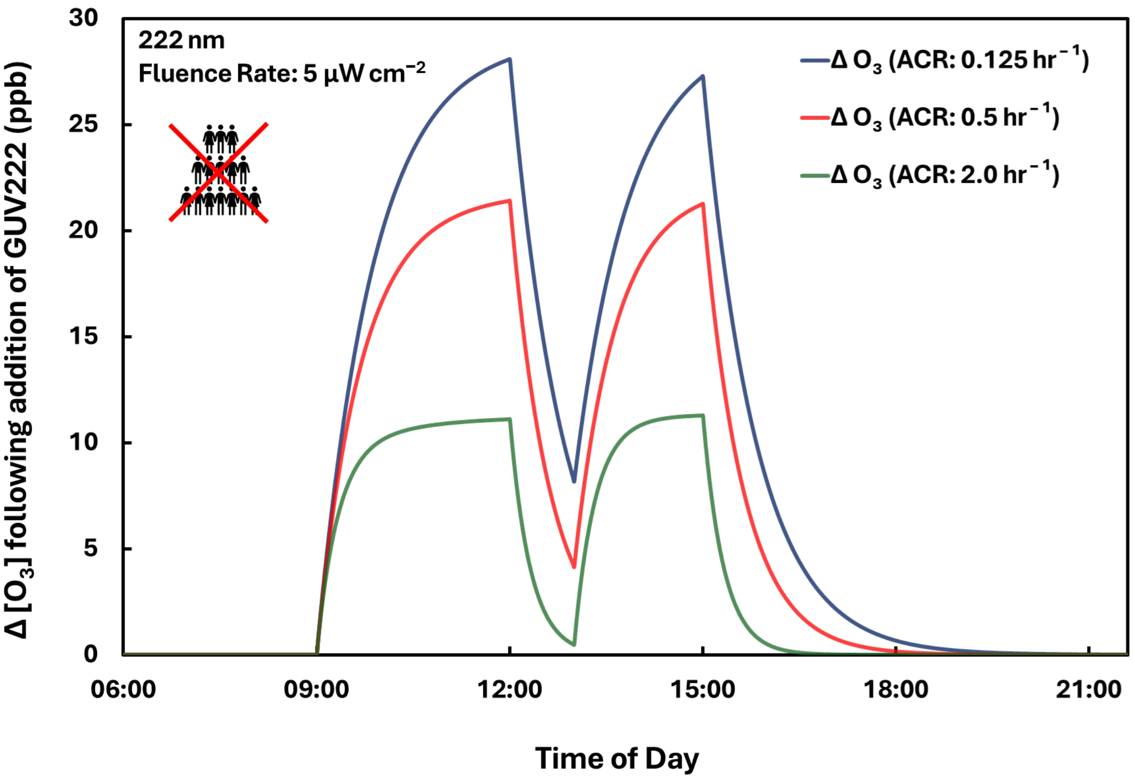


Figure S7: The difference in the mixing ratio of ozone in an unoccupied classroom at varying air change rates following the addition of a GUV222 5 $\mu$W cm$^{-2}$ lamp.

# References


Z. Wang, D. Shaw, T. Kahan, C. Schoemaecker and N. Carslaw, A modeling study of the impact of photolysis on indoor air quality, *Indoor Air*, 2022, **32**, 1–10.

D. R. Shaw, T. J. Carter, H. L. Davies, E. Harding-Smith, E. C. Crocker, G. Beel, Z. Wang and N. Carslaw, INCHEM-Py v1.2: a community box model for indoor air chemistry, *Geoscientific Model Development*, 2023, **16**, 7411–7431.

E. Eadie, W. Hiwar, L. Fletcher, E. Tidswell, P. O'Mahoney, M. Buonanno, D. Welch, C. S. Adamson, D. J. Brenner, C. Noakes and K. Wood, Far-UVC (222 nm) efficiently inactivates an airborne pathogen in a room-sized chamber, *Scientific Reports*, 2022, **12**, 4373.

Z. Peng, D. A. Day, G. A. Symonds, O. J. Jenks, H. Stark, A. V. Handschy, J. A. de Gouw and J. L. Jimenez, Significant Production of Ozone from Germicidal UV Lights at 222 nm, *Environmental Science and Technology Letters*, 2023, **10**, 668–674.

T. J. Carter, D. G. Poppendieck, D. Shaw and N. Carslaw, A Modelling Study of Indoor Air Chemistry: The Surface Interactions of Ozone and Hydrogen Peroxide, *Atmospheric Environment*, 2023, **297**, 119598.

T. J. Carter, D. R. Shaw, D. C. Carslaw and N. Carslaw, Indoor cooking and cleaning as a source of outdoor air pollution in urban environments, *Environmental Science: Processes & Impacts*, 2024, **26**, 975–990.

S. Uchiyama, T. Tomizawa, A. Tokoro, M. Aoki, M. Hishiki, T. Yamada, R. Tanaka, H. Sakamoto, T. Yoshida, K. Bekki, Y. Inaba, H. Nakagome and N. Kunugita, Gaseous chemical compounds in indoor and outdoor air of 602 houses throughout Japan in winter and summer, *Environmental Research*, 2015, **137**, 364–372.

A. Baudic, V. Gros, S. Sauvage, N. Locoge, O. Sanchez, R. Sarda-Estève, C. Kalogridis, J. E. Petit, N. Bonnaire, D. Baisnée, O. Favez, A. Albinet, J. Sciare and B. Bonsang,

Seasonal variability and source apportionment of volatile organic compounds (VOCs) in the Paris megacity (France), *Atmospheric Chemistry and Physics*, 2016, **16**, 11961–11989.

H. Lü, S. Wen, Y. Feng, X. Wang, X. Bi, G. Sheng and J. Fu, Indoor and outdoor carbonyl compounds and BTEX in the hospitals of Guangzhou, China, *Science of the Total Environment*, 2006, **368**, 574–584.

S. Mentese and B. Bas, A year-round motoring of ambient volatile organic compounds across Dardanelles strait, *Journal of Chemical Metrology*, 2020, **14**, 177–189.

M. A. Bari and W. B. Kindzierski, Ambient volatile organic compounds (VOCs) in Calgary, Alberta: Sources and screening health risk assessment, *Science of The Total Environment*, 2018, **631-632**, 627–640.

A. Sturaro, R. Rella, G. Parvoli and D. Ferrara, Long-term phenol, cresols and BTEX monitoring in urban air, *Environmental Monitoring and Assessment*, 2010, **164**, 93–100.

M. A. Bari, W. B. Kindzierski and D. Spink, Twelve-year trends in ambient concentrations of volatile organic compounds in a community of the Alberta Oil Sands Region, Canada, *Environment International*, 2016, **91**, 40–50.

E. Gallego, F. J. Roca, J. F. Perales, X. Guardino, E. Gadea and P. Garrote, Impact of formaldehyde and VOCs from waste treatment plants upon the ambient air nearby an urban area (Spain), *Science of The Total Environment*, 2016, **568**, 369–380.

L. S. Brickus, J. N. Cardoso and F. R. De Aquino Neto, Distributions of indoor and outdoor air pollutants in Rio de Janeiro, Brazil: Implications to indoor air quality in bayside offices, *Environmental Science and Technology*, 1998, **32**, 3485–3490.

H. Hellén, A. P. Praplan, T. Tykkä, I. Ylivinkka, V. Vakkari, J. Bäck, T. Petäjä, M. Kulmala and H. Hakola, Long-term measurements of volatile organic compounds highlight

the importance of sesquiterpenes for the atmospheric chemistry of a boreal forest, *Atmospheric Chemistry and Physics*, 2018, **18**, 13839–13863.

H. Hakola, H. Hellén, V. Tarvainen, J. Bäck, J. Patokoski and J. Rinne, Annual variations of atmospheric VOC concentrations in a boreal forest, *Boreal Environment Research*, 2009, **14**, 722–730.

S. Z. He, Z. M. Chen, X. Zhang, Y. Zhao, D. M. Huang, J. N. Zhao, T. Zhu, M. Hu and L. M. Zeng, Measurement of atmospheric hydrogen peroxide and organic peroxides in Beijing before and during the 2008 Olympic Games: Chemical and physical factors influencing their concentrations, *Journal of Geophysical Research: Atmospheres*, 2010, **115**, D17307.

E. Dlugokencky, *NOAA/GML CH4 Trends, https://gml.noaa.gov/ccgg/trends/ (Date Accessed: March 2022)*, 2022, `https://gml.noaa.gov/webdata/ccgg/trends/ch4/ch4_mm_gl.txt`.

F. Vichi, L. Mašková, M. Frattoni, A. Imperiali and J. Smolík, Simultaneous measurement of nitrous acid, nitric acid, and nitrogen dioxide by means of a novel multipollutant diffusive sampler in libraries and archives, *Heritage Science*, 2016, **4**, 4.

L. Liu, X. Wang, J. Chen, L. Xue, W. Wang, L. Wen, D. Li and T. Chen, Understanding unusually high levels of peroxyacetyl nitrate (PAN) in winter in Urban Jinan, China, *Journal of Environmental Sciences (China)*, 2018, **71**, 249–260.

M. Li, E. Karu, C. Brenninkmeijer, H. Fischer, J. Lelieveld and J. Williams, Tropospheric OH and stratospheric OH and Cl concentrations determined from CH4, CH3Cl, and SF6 measurements, *npj Climate and Atmospheric Science*, 2018, **1**, 29.

EEA, *European Air Quality Portal, https://eeadmz1-cws-wp-air02.azurewebsites.net/, (Date Accessed: December 2021)*, 2018.

H. Plaisance, J. Vignau-Laulhere, P. Mocho, N. Sauvat, K. Raulin and V. Desauziers, Volatile organic compounds concentrations during the construction process in newly-built timber-frame houses: Source identification and emission kinetics, *Environmental Science: Processes and Impacts*, 2017, **19**, 696–710.

T. Alapieti, E. Castagnoli, L. Salo, R. Mikkola, P. Pasanen and H. Salonen, The effects of paints and moisture content on the indoor air emissions from pinewood (Pinus sylvestris) boards, *Indoor Air*, 2021, **31**, 1563–1576.

Y. H. Cheng, C. C. Lin and S. C. Hsu, Comparison of conventional and green building materials in respect of VOC emissions and ozone impact on secondary carbonyl emissions, *Building and Environment*, 2015, **87**, 274–282.

M. Kruza and N. Carslaw, How do breath and skin emissions impact indoor air chemistry?, *Indoor Air*, 2019, **29**, 369–379.
